# Strategic Single-Residue Substitution in the Antimicrobial Peptide Esc(1–21) Confers Activity against *Staphylococcus aureus*, Including Drug-Resistant and Biofilm Phenotype

**DOI:** 10.1021/acsinfecdis.4c00130

**Published:** 2024-06-07

**Authors:** Maria Rosa Loffredo, Bruno Casciaro, Rosa Bellavita, Cassandra Troiano, Diego Brancaccio, Floriana Cappiello, Francesco Merlino, Stefania Galdiero, Giancarlo Fabrizi, Paolo Grieco, Lorenzo Stella, Alfonso Carotenuto, Maria Luisa Mangoni

**Affiliations:** † Department of Biochemical Sciences, Laboratory Affiliated to Istituto Pasteur Italia-Fondazione Cenci Bolognetti, Sapienza University of Rome, 00185 Rome, Italy; ‡ Department of Pharmacy, University of Naples “Federico II”, 80131 Naples, Italy; § Department of Chemical Science and Technologies, University of Rome Tor Vergata, 00133 Rome, Italy; ∥ Department of Chemistry and Technology of Drugs, “Department of Excellence 2018−2022”, Sapienza University of Rome, 00185 Rome, Italy

**Keywords:** antimicrobial peptides, *Staphylococcus aureus*, bent helical structure, α-aminoisobutyric acid, biofilm, multidrug-resistant strains

## Abstract

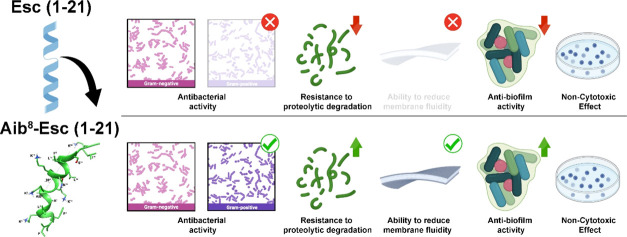

*Staphylococcus aureus*, a bacterium resistant to multiple drugs, is a significant cause of illness and death worldwide. Antimicrobial peptides (AMPs) provide an excellent potential strategy to cope with this threat. Recently, we characterized a derivative of the frog-skin AMP esculentin-1a, Esc(1–21) (**1**) that is endowed with potent activity against Gram-negative bacteria but poor efficacy against Gram-positive strains. In this study, three analogues of peptide **1** were designed by replacing Gly^8^ with α-aminoisobutyric acid (Aib), Pro, and dPro (**2**–**4**, respectively). The single substitution Gly^8^ → Aib^8^ in peptide **2** makes it active against the planktonic form of Gram-positive bacterial strains, especially *Staphylococcus aureus*, including multidrug-resistant clinical isolates, with an improved biostability without resulting in cytotoxicity to mammalian cells. Moreover, peptide **2** showed a higher antibiofilm activity than peptide **1** against both reference and clinical isolates of *S*. *aureus*. Peptide **2** was also able to induce rapid bacterial killing, suggesting a membrane-perturbing mechanism of action. Structural analysis of the most active peptide **2** evidenced that the improved biological activity of peptide **2** is the consequence of a combination of higher biostability, higher α helical content, and ability to reduce membrane fluidity and to adopt a distorted helix, bent in correspondence of Aib^8^. Overall, this study has shown how a strategic single amino acid substitution is sufficient to enlarge the spectrum of activity of the original peptide **1**, and improve its biological properties for therapeutic purposes, thus paving the way to optimize AMPs for the development of new broad-spectrum anti-infective agents.

The list of the most dangerous bacterial pathogens, compiled by the World Health Organization, includes the Gram-positive bacterium *Staphylococcus aureus*, which is a regular component of the normal human flora, generally located on the skin and nose.^1,2^ This bacterium does not normally cause health problems, but if it enters the bloodstream or if it meets internal tissues like lungs and heart, invading host cells, it can provoke serious infections, especially in hospital-acquired settings, giving rise to a large variety of clinical manifestations.^2,3^ Treatment of such infections usually involves antibiotics and cleaning of the damaged area. However, several *S*. *aureus* strains no longer respond, and have become resistant to the available antibiotics such as methicillin-resistant *Staphylococcus aureus* (MRSA).^4^ Therefore, the design of antimicrobial agents that are distinct in their mode of action from conventional antibiotics is essential. Naturally occurring cationic antimicrobial peptides (AMPs) are produced by all organisms of the evolutionary scale as key effectors of the innate immune system and hold great promise for the development of novel drugs to tackle microbial infections, including those provoked by multidrug-resistant (MDR) bacteria.^5,6^ This is mostly due to their primary mechanism of action that generally involves interaction with the anionic membranes of the target microbes, followed by its destabilization, through pore formation or disintegration, thus limiting the evolution of resistant microorganisms.^7^ Furthermore, AMPs have been defined as “dirty drugs”, due to the existence of additional mechanisms addressing cytoplasmic targets, especially at sublethal doses.^8^

Among natural sources of AMPs, amphibian skin is particularly rich in these molecules.^9,10^ Esculentin-1 peptides were first discovered in the skin secretion of frogs from the *Pelophylax lessonae/ridibundus* genus (known as *Rana esculenta*)^11,12^ and later from the skin of other closely related species.^13,14^ Previous studies performed with 1–18 moieties of esculentin-1b AMP, amidated at its C-terminus, namely Esc-1b(1–18), (GIFSKLAGKKLKNLLISG-NH_2_), showed that it is endowed with similar antibacterial activity to that of its full-length parent peptide, mainly toward Gram-negative bacteria.^12,15^ Structural investigation using nuclear magnetic resonance (NMR) spectroscopy in negatively charged lipid micelles, to mimic the bacterial membrane, revealed that the peptide adopted an α-helical conformation throughout its entire length. However, there was a flexible kink at the level of Gly^8^ which divides the peptide sequence into two separate helical segments.^16^ It is worth noting that the minimum length required for an α-helical peptide to span the phospholipid bilayer of membranes is approximately 20 amino acids. Based on this information, a longer isoform of the peptide was synthesized and characterized for its biological properties.^17−20^ This isoform, known as Esc(1–21) consisted of the first 20 residues of esculentin-1a, with the addition of amidated Gly (Table 1).^17−20^ Note that in comparison to Esc-1b(1–18), the longer peptide Esc(1–21) carries the substitution Leu^11^ → Ile^11^. Esc(1–21) was more active than Esc-1b(1–18), but with limited efficacy against Gram-positive bacterial strains.^21,22^ Interestingly, the introduction of a nonproteinogenic amino acid, i.e., the α-aminoisobutyric acid (Aib) at three different positions (1, 10, and 18) in the primary structure of Esc(1–21) was found to stabilize the helical content of the peptide, making it active against Gram-positive bacteria including *S*. *aureus*. Nevertheless, the peptide analogue bearing Aib residues was cytotoxic to human cells at concentrations higher than 4 μM, corresponding to the minimal peptide dosage which inhibits bacterial growth.^23^

**Table 1:** Peptide Sequences of Esc(1-21) and Its Gly8-Replaced Analogues^a^

peptide	name	sequence
**1**	Esc(1–21)	GIFSKLAGKKIKNLLISGLKG-NH_2_
**2**	[Aib^8^]-Esc(1–21)	GIFSKLA**Aib**KKIKNLLISGLKG-NH_2_
**3**	[Pro^8^]-Esc(1–21)	GIFSKLA**P**KKIKNLLISGLKG-NH_2_
**4**	[dPro^8^]-Esc(1–21)	GIFSKLA***p***KKIKNLLISGLKG-NH_2_

a Residue variations compared to **1** are in bold. Residues in d-configuration are lowercase.

Taking advantage of such observations, with the aim to (i) modulate the helicity of Esc(1–21), which is expected to influence the peptide’s spectrum of activity against Gram-positive bacterial species^23^ and (ii) to potentiate its resistance to proteolytic degradation without enhancing the noxious effect toward human cells, we designed novel analogues of Esc(1–21) by replacing a single residue, i.e., Gly^8^ with the non-natural helicogenic amino acid Aib, the helical kink-inducing Pro and its enantiomer dPro.

We investigated the biological activity, proteolytic stability, and biophysical characterization of the mechanism of action of the three analogues through microbiological and fluorescent dye-based assays, as well as their structural characterization in model membranes, by circular dichroism (CD) spectroscopy and NMR techniques.

## Results

### Design and Synthesis

Esc(1–21) (peptide **1**, Table 1) was found to adopt a straight and amphipathic α-helical conformation in membrane mimetic solution.^18^ At variance, its close analogue Esc-1b(1–18) was shown to possess a kinked helix on Gly^8^ in similar media.^16^ To explore the effect of the modulation of this α-helix on the antimicrobial activity, we designed novel analogues of Esc(1–21) by replacing Gly^8^ with an (i) Aib residue, which stabilizes an α-helix or a 3_10_ helix (peptide **2**);^24,25^ (ii) a Pro residue, which is generally found in correspondence with kinks in transmembrane helices (peptide **3**);^26^ and (iii) a dPro residue which generally induces significant disruption in the α-helical structure (peptide **4**). Variations of physicochemical properties such as amphipathicity and hydrophobicity after these substitutions could have played a role in antimicrobial activity of the resulting peptides. Moreover, the replacement of the single residue Gly^8^ with the non-natural amino acids should potentiate peptide resistance to proteolytic degradation.

Peptides **1**–**4** were synthesized by combining ultrasound chemistry and solid-phase peptide (US-SPPS) methodology as reported elsewhere.^27^ Upon the achievement of crude peptides, these were purified and analyzed by reversed-phase high-pressure liquid chromatography (RP-HPLC) and tested for their purity (>95%, see the Supporting Information, Figures S1–S4). Their identity was confirmed through electrospray ionization mass spectrometry (Supporting Information, Table S1 and Figures S5–S8).

### Antimicrobial Activity

The antimicrobial activity of Esc(1–21) analogues was assessed by the broth microdilution method to determine the minimal inhibitory concentration (MIC) against a panel of Gram-negative and Gram-positive bacterial strains. MIC values were compared to those of parent peptide **1** (Table 2).

**Table 2:** Antimicrobial Activity of Peptides **1**–**4^a^**

	MIC (μM)			
	1	2	3	4
**Gram-Negative**				
*E*. *coli* ATCC 25922	1.56	0.78	3.12	6.25
*A*. *baumannii* ATCC 19606	1.56	0.78	1.56	12.5
*P*. *aeruginosa* ATCC 25853	3.12	3.12	6.25	25
**Gram Positive**				
*S*. *epidermidis* ATCC 12228	3.12	0.78	6.25	12.5
*S*. *aureus* ATCC 25923	>100	12.5	>100	>100
*S*. *aureus* MDR #1	>100	12.5	>100	>100
*S*. *aureus* MDR #2	100	12.5	>100	>100
*S*. *aureus* MDR *#3*	100	12.5	>100	>100
*S*. *aureus* MDR *#4*	>100	6.25	>100	>100

a MICs are obtained from three identical readings of four independent experiments.

As reported in Table 2, peptides **3** and **4** in which Gly8 was replaced with Pro and dPro, respectively, were found to be ∼2 to 8-fold less active than the parent peptide **1** against all the tested microorganisms. Like peptide **1**, they did not show any antibacterial effect against *S*. *aureus* (MIC ≥ 100 μM). On the contrary, peptide **2**, containing the Aib residue at position 8 of the sequence, showed the strongest activity against the Gram-positive *S*. *epidermidis* strain, and it was the only one exerting activity against the Gram-positive *S*. *aureus* ATCC 25923 (MIC = 12.5 μM). The activity against Gram-negative bacteria was similar to that of peptide **1**. Importantly, the anti-*S*. *aureus* activity was also confirmed against four multidrug-resistant clinical isolates (resistance profile in the Supporting Information, Table S2), against which peptide **2** had a MIC ranging from 6.25 to 12.5 μM.

**Figure 1:**
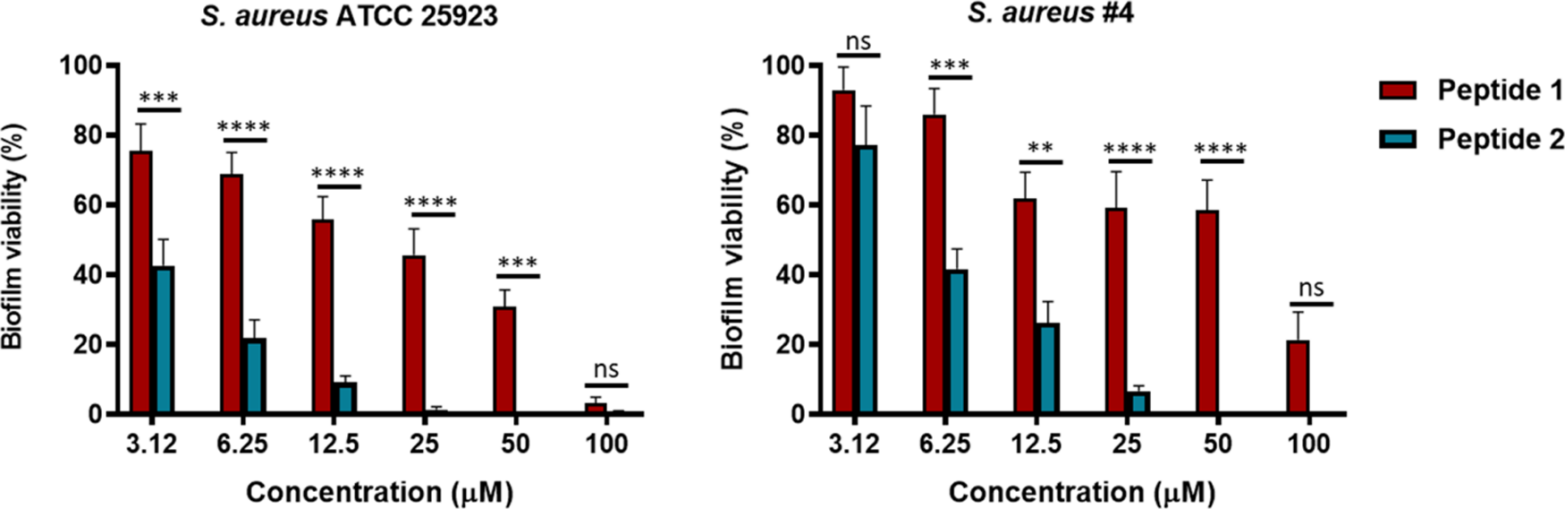
Activity of peptides **1** and **2** against the biofilm of *S*. *aureus* ATCC 25923 and *S*. *aureus* #4, after 2 h of treatment. Biofilm viability was evaluated by measuring the reduction of MTT to its insoluble formazan (as reported in the Materials and Methods section) and expressed as percentage compared to that of untreated samples (bacterial biofilm not treated with the peptide, 100% viability). Data are the mean ± standard error of the mean (SEM) of three independent experiments performed in duplicate. Statistical analysis was conducted using two-way ANOVA to determine the significance between the two peptides. **, *p* < 0.01; ***, *p* < 0.001; ****, *p* < 0.0001; ns, not significant.

Moreover, the activity of peptide **2** against *S*. *aureus* was retained against the sessile form of this bacterium, the ability of the peptide to kill 20-h preformed *S*. *aureus* biofilms was investigated and compared to that of the wild-type peptide, by the 3-(4,5-dimethylthiazol-2-yl)-2,5-diphenyltetrazolium bromide (MTT) assay, 2 h after peptide addition at different concentrations. Either the reference strain of *S*. *aureus* (ATCC 25923) or a representative MDR clinical isolate (*S*. *aureus* #4) were used. Remarkably, peptide **1** showed a weaker antibiofilm activity than the Aib-containing analogue, at all concentrations tested, in agreement with its lower potency toward the planktonic form of these microorganisms (Table 2). In fact, as reported in Figure 1, peptide **1** was able to reduce ∼90% of biofilm viability only when it was assayed at 100 μM against the reference strain of *S*. *aureus* ATCC 25923. On the contrary, peptide **2** was able to reduce more than 90% biofilm viability of both reference and clinical isolates at concentrations of 25 μM, which are only 2- or 4-fold higher than the corresponding MIC against the two strains.

### Peptide’s Effect on Cell Viability

To investigate whether the introduction of one unnatural amino acid at position 8 in the primary structure of Esc(1–21) affected the viability of mammalian cells, the cytotoxicity of peptide **2** was evaluated by the MTT assay on human immortalized keratinocytes (HaCaT cells). Keratinocytes are the principal cell type in the epidermis, the outermost layer of the skin, and are easily infected by *S*. *aureus* in the presence of lesions or following reduced host immune defenses.^28−30^

**Figure 2:**
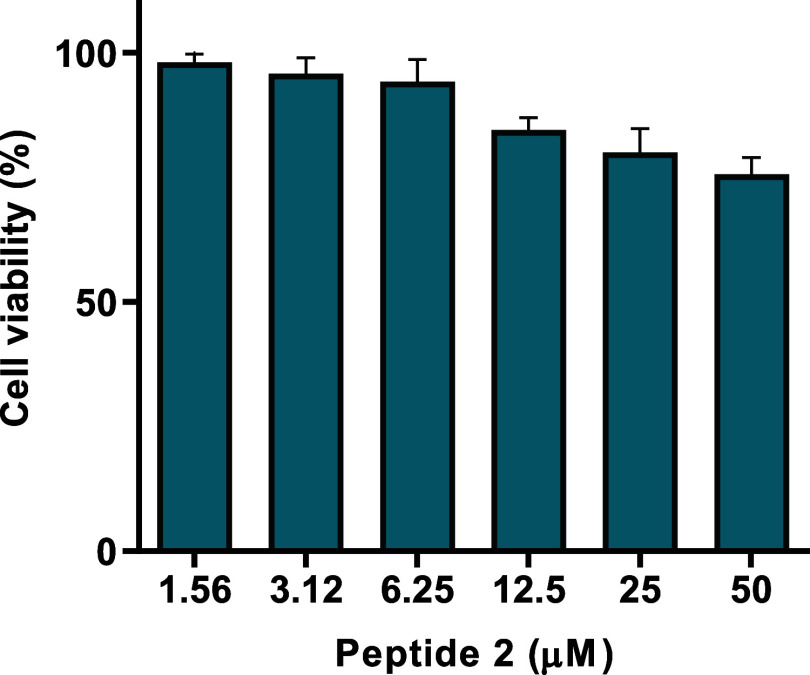
Effect of peptide **2** on the survival of HaCaT cells was assessed using the MTT assay after 24 h of treatment. The results are presented as percentage compared to the untreated control cells and represent the mean of three independent experiments ± SEM.

As reported in Figure 2, peptide **2** did not induce any significant cytotoxic effect up to a concentration of 25 μM after 24 h of treatment, while at the highest peptide concentration tested (i.e., 50 μM) it provoked a slight reduction (∼20%) of metabolically active cells. This result was comparable to that previously described for the parent peptide **1**, whose lethal dose causing 50% killing (LD_50_) was found to be >50 μM.^20^

### Peptide Stability in Serum

**Figure 3:**
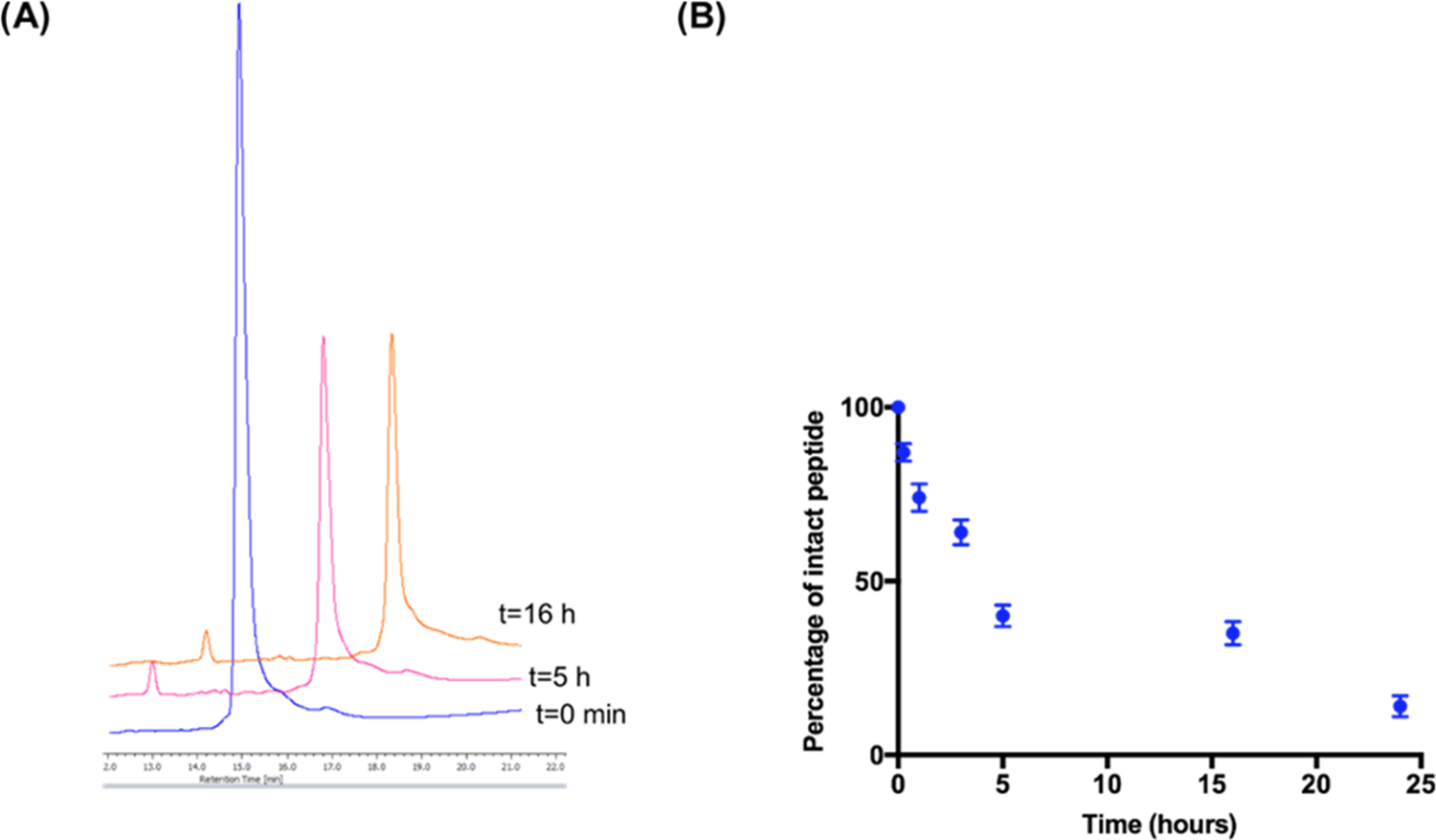
Stability of peptide **2** in 50% fresh bovine serum at different incubation times at 37 °C. (A) The panel reports the most representative RP-HPLC chromatograms of peptide **2** at 0, 5, and 16 h. (B) The panel reports the percentage of nondegraded peptide (%) after 1, 3, 4, 5, 16, and 24 h. Data represent the mean ± standard deviation (SD) of three independent experiments.

Considering the potential development of AMPs as suitable therapeutic agents to fight infections, we studied the stability of the most promising peptide **2** in biological fluids. For this purpose, the amount of intact peptide was monitored within 24 h of incubation at 37 °C in the presence of 50% fresh bovine serum (Figure 3).

The data showed that the percentage of intact peptide **2** was ∼90%, ∼75%, and ∼65% after 1, 3, and 4 h of incubation, respectively. Interestingly, after 5 h treatment with 50% bovine serum, the nondegraded amount of peptide was ∼40% and the same percentage of intact peptide was detected even after 16 h. Instead, the peptide was further degraded after 24 h, leaving only ∼15% of the intact peptide.

### Biological Characterization of the Mechanism of Action of Peptide **2**

#### Cytoplasmic Membrane Perturbation

Perturbation of the cytoplasmic membrane has already been reported to be the principal mechanism of the antimicrobial activity of parent peptide **1** against Gram-negative bacteria. To verify whether the active peptide **2** had a membrane-perturbing effect against Gram-positive bacteria, the fluorescent probe Sytox Green was employed to carry out fluorescence studies on two different bacterial strains *e*.*g*. *S*. *aureus* ATCC 25923 and *S*. *epidermidis* ATCC 12228 to measure the membrane perturbation induced by the peptide during the first 30 min from its addition at different concentrations, from 0.39 to 12.5 μM. The results were compared to those of untreated control cells.

**Figure 4:**
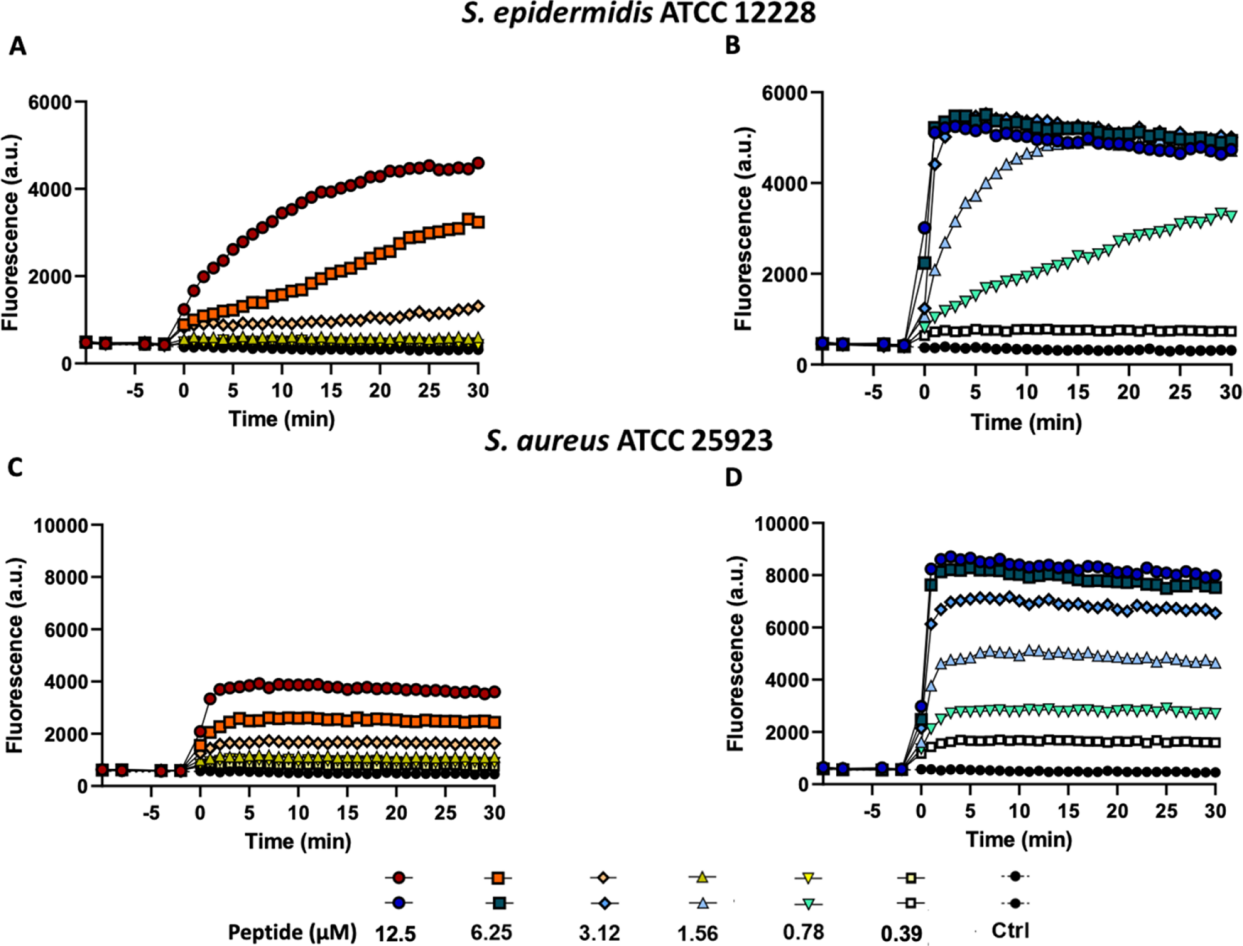
Kinetics of cytoplasmic membrane permeabilization of *S*. *epidermidis* ATCC 12228 and *S*. *aureus* ATCC 25923 induced by the addition of peptide **1** (panels A and C) and peptide **2** (panels B and D) at different concentrations. Alterations of the permeability of the cytoplasmic membrane allowed the Sytox Green probe (1 μM) to enter the cell and bind intracellular nucleic acids, resulting in an increase of fluorescence intensity. Controls (Ctrl) are microbial cells without the addition of any peptide. The reported values are from one representative experiment out of three.

As reported in Figure 4 (panels B and D), peptide **2** induced a fast and dose-dependent membrane perturbation process. Within the first minutes from its addition, the highest values of fluorescence intensity were recorded for both bacterial strains, in the concentration ranges of 1.56–3.12 and 6.25–12.5 μM for *S*. *epidermidis* and *S*. *aureus*, respectively. In comparison, a much weaker membrane perturbation was found for peptide **1** (Figure 4, panels A and C), especially against *S*. *aureus*, according to its lower antimicrobial activity.

#### Killing Kinetics against *S. aureus* ATCC 25923

To get insight into the ability of the most active peptide **2** to exert a bactericidal activity against *S*. *aureus* cells, its effect on the viability of *S*. *aureus* ATCC 25923 was assessed by counting the number of colony-forming units (CFU) during 120 min of exposure of bacterial cells to different peptide concentrations, corresponding to the 2 × MIC (i.e., 25 μM), MIC (i.e., 12.5 μM), 1/2 × MIC (i.e., 6.25 μM), and 1/4 × MIC (3.12 μM).

**Figure 5:**
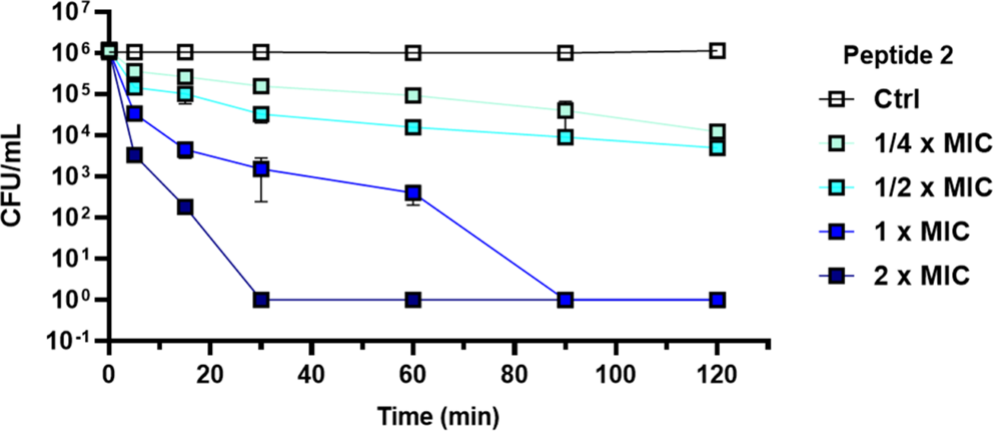
Effect of peptide **2** on *S*. *aureus* ATCC 25923 cell viability. Bacteria (1 × 10^6^ CFU/mL) were incubated with peptide **2** at 2 × MIC (25 μM), MIC (12.5 μM), 1/2 × MIC (6.25 μM), and 1/4 × MIC (3.12 μM) in phosphate-buffered saline (PBS) at 37 °C. The number of surviving cells (CFU/mL) was calculated at different time points (5, 15, 30, 60, 90, and 120 min). Data represent the mean ± SD of four independent experiments.

As shown in Figure 5, peptide **2** affected the viability of *S*. *aureus* cells in a dose-dependent manner.

Interestingly, it induced a significant and rapid reduction in CFU within a few minutes when used at 2 × MIC (25 μM). Indeed, a decrease of more than 99% in viable bacterial cells was recorded after 5 min and more than 99.9% after 15 min from peptide addition, with total cell mortality after 30 min. In comparison, when peptide **2** was used at lower concentrations, i.e., 1/4 × MIC, 1/2 × MIC, and MIC it caused ∼1 log (90%), ∼ 2 log (99%), and ∼3 log (99,9%) reduction in the number of CFUs, respectively, after 1 h.

### Biophysical Characterization of the Mechanism of Action

#### Peptide Aggregation

To further expand our knowledge of the mechanism of action of the three analogues, we studied their aggregation. First, we monitored the tendency of peptides to aggregate in solution, in a range of concentrations from 0.8 to 200 μM by using Nile Red as the fluorophore. Under these conditions, peptide **1** showed a slight tendency to aggregate in solution, with a calculated critical aggregation concentration (CAC) of 70 μM, while peptides **2** and **3** did not produce any blue shift in the Nile Red fluorescence spectrum, indicating the presence of a monomeric state (data not shown). Notably, all peptides are in a monomeric state under the experimental biological conditions.

**Figure 6:**
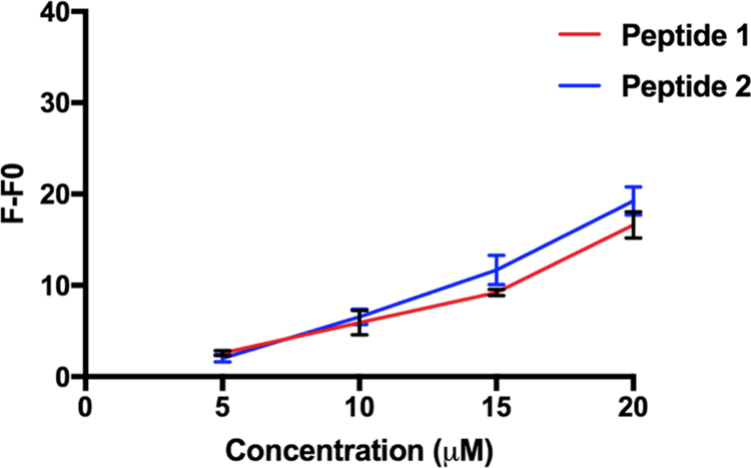
ThT fluorescence as a function of the peptide concentration of **1** and **2** in liposomes mimicking Gram-positive bacterial membranes (POPG/POCL, 6:4, *mol:mol*) (100 μM). On the *y*-axis, *F* indicates the value of fluorescence after peptide addition, while *F*_0_ represents the initial fluorescence in the absence of peptide. Data represent the mean ± SD of three independent experiments.

The tendency to aggregate in membranes was explored by using large unilamellar vesicles (LUVs) mimicking the composition of the membrane of Gram-positive bacteria [(1-palmitoyl-2-oleoyl-*sn*-glycero-3-phospho-(1′-RAC-glycerol)(POPG)/(1′,3′-bis[1-palmitoyl-2-oleoyl-*sn*-glycero-3-phospho]-glycerol) (POCL), 6:4, *mol:mol*)] and thioflavin T (ThT) as the fluorescent probe.^31^ The aggregation results obtained in LUVs are reported in Figure 6.

A dramatic increase in fluorescence as a function of concentration was observed for peptides **1** and **2**, suggesting a progressive phenomenon of aggregation in LUVs. In contrast, peptide **3** did not show aggregation at any peptide concentration (data not shown).

#### Peptide Interaction with the Gram-Positive Membrane Model

The interaction between peptides and LUVs mimicking Gram-positive bacterial membranes was evaluated by measuring the changes in the zeta potential and mean diameter of liposomes and by evaluating the membrane fluidity changes after treatment with each peptide by using Laurdan as the fluorescent probe.

Interestingly, after treatment of LUVs with each peptide, the zeta potential changed significantly due to strong electrostatic interactions established between LUVs and peptides. In fact, the zeta potential of LUVs switched from a negative value of −42 ± 2 mV to highly positive values (Table 3). We measured the values of +10.9 ± 0.1, + 13 ± 2, and +15 ± 2 mV for peptides **1**, **2**, and **3**, respectively. Moreover, the values of the polydispersity index (PDI) before and after the peptide treatment are also reported in Table 3. PDI values of 0.2 and below are indicative of a monodisperse colloidal solution.^32^

**Table 3:** Values of Zeta Potential and Mean Diameter of LUVs after Treatment with Peptides **1**, **2**, and **3^a^**

compound	zeta potential (mV)	mean diameter (nm)	mean PDI
unloaded LUVs	–42 ± 2	107 ± 1	0.17 ± 0.01
LUVs + peptide **1**	+10.9 ± 0.1	175 ± 1	0.18 ± 0.02
LUVs + peptide **2**	+13 ± 2	175 ± 2	0.16 ± 0.01
LUVs + peptide **3**	+15 ± 2	138 ± 4	0.28 ± 0.05

a Data represent the mean ± SD of three independent experiments.

Moreover, after the treatment of LUVs with each peptide, we also observed variations in the liposome size, as reported in Table 3. In the absence of the peptide, we measured a mean diameter of 107 ± 1 nm for the LUVs, while their mean diameter considerably changed after the treatment with each peptide. In particular, we observed a significant increase in the presence of the peptides **1** (175 ± 1 nm) and **2** (175 ± 2 nm), probably due to the ability of these peptides to induce LUV fusion. In contrast, in the presence of peptide **3**, a slight change in mean diameter of 138 ± 4 nm was measured.

In addition, the effect of peptides **1**–**3** on the membrane fluidity was investigated by using Laurdan-labeled LUVs. The emission of the Laurdan probe can shift from 440 nm, indicating a membrane ordered phase, to 490 nm, when the bilayer is in the disordered phase.^33^ The change in the fluidity of the bilayer was calculated by determining the generalized polarization (GP) parameter at a peptide concentration of 20 μM. As reported in Table 4, after the treatment, peptide **2** caused a slight but significant decrease in lipid fluidity (GP went from −0.51 ± 0.01 to −0.45 ± 0.01). Notably, peptides **1** and **3** did not change the lipid order and dynamics.

**Table 4:** Membrane Fluidity Evaluation Using the GP Value^a^

compound	GP value
unloaded LUVs	–0.51 ± 0.01
LUVs + peptide **1** (20 μM)	–0.50 ± 0.01
LUVs + peptide **2** (20 μM)	–0.45 ± 0.02
LUVs + peptide **3** (20 μM)	–0.51 ± 0.02

a Data represent the mean ± SD of three independent experiments.

#### Effect of Peptide **2** on Dye Leakage

To further expand our knowledge about the membranolytic mechanism of peptide **2** and the extent of peptide-induced membrane damage, we used artificial LUVs, mimicking the composition of the membrane of Gram-positive bacteria loaded with the fluorescent probe carboxyfluorescein (CF). The purpose of this study was to address the membrane destabilization property of peptide **2**, by measuring the dye leakage from LUVs upon peptide addition.

**Figure 7:**
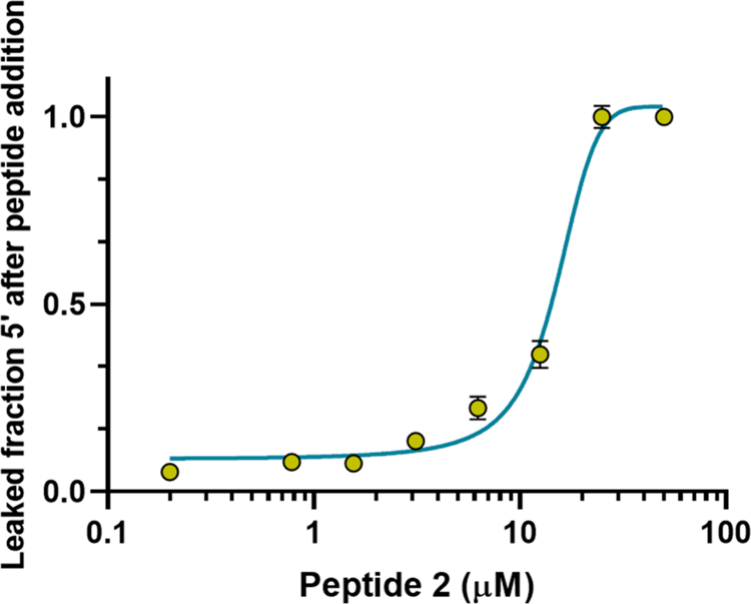
Effect of different concentrations of peptide **2** on the leakage of CF encapsulated into POPG/POCL (6:4, *mol:mol)* LUVs after 5 min. LUVs were used at a final lipid concentration of 100 μM. Data points are the mean ± SD of three different experiments.

As indicated in Figure 7, a fast membrane-perturbing activity was displayed by analogue **2** with a total CF leakage within 5 min of its addition to the LUVs at 25 μM, while the 12.5 μM peptide caused a leakage of about 40%.

### Structural Characterization of Peptide 2

The secondary structure of promising peptide **2** was determined by CD and NMR spectroscopy in the presence of artificial membranes mimicking the lipid composition of the cytoplasmic membrane of Gram-positive bacteria. Peptides **1** and **3** were also investigated for comparison.

#### Circular Dichroism Analysis

**Figure 8:**
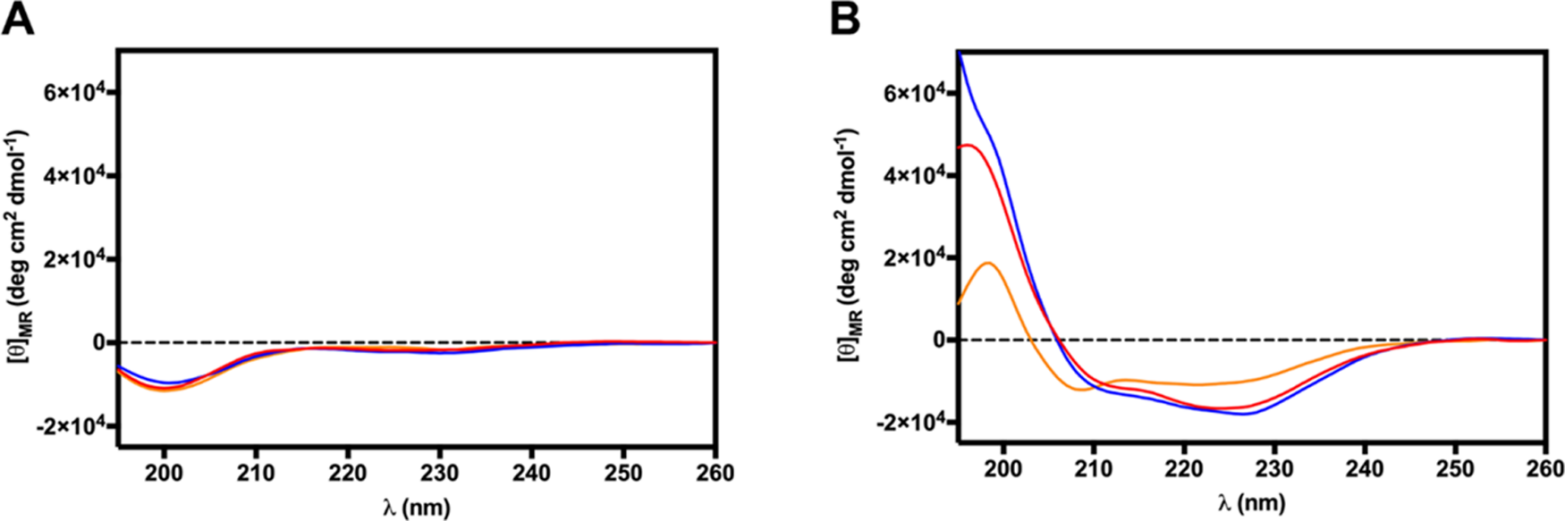
Circular dichroism spectra of peptides **1** (red line), **2** (blue line), and **3** (orange line) at 20 μM measured in (A) water and (B) in the presence of POPG/POCL (6:4 *mol/mol*) LUVs (500 μM).

The secondary structure of peptides **1**–**3** was initially characterized by CD spectroscopy in water and in the presence of POPG/POCL LUVs (peptide 20 μM, lipid 500 μM) (Figure 8). All three peptides are mostly unstructured in water (with a minimum at about 200 nm), as expected for such short AMPs. The lower minimum of **2** at 200 nm indicates that this peptide can be slightly more structured in water than parent peptide **1** and analogue **3**. In the presence of liposomes mimicking the Gram-positive bacterial membrane, the CD spectra were characteristic of a helical structure showing two minima at around 208 and 222 nm. The intensity ratio between the two minima at 222 and 208 nm was greater than 1.0 for peptides **1** and **2**, indicating a helical conformation in its oligomeric state.^34^ In contrast, that ratio was <1.0 for peptide **3** pointing out its monomeric state in the lipid environment. The secondary structure content of the peptides in LUVs was predicted using the online server for protein secondary structure analyses, Bestsel.^35^ The prediction indicates that the helical content in the three peptides increases in the order **3** < **1** < **2** (Table 5).

**Table 5:** Percentages of Secondary Structure of Peptides **1**–**3** in POPG/POCL (6:4, *mol/mol*) LUVs Calculated Using Bestsel

peptide	% helix	% β-sheet	% turn	% others
**1**	51.1	2.0	9.6	37.3
**2**	59.1	3.9	9.1	27.9
**3**	45.1	2.9	7.7	44.3

#### NMR Analysis with POPG/POCL Bicelles

The structure of analogue **2** was also investigated by NMR spectroscopy in the presence of isotropic bicelles composed of POPG/POCL (6:4, *mol*/*mol*) as long-chain phospholipid and 1,2-dihexanoyl-*sn*-glycero-3-phosphocholine (DHPC) as detergent (*q* = 0.1, *C*_L_ = 9%), and compared to that of the parent peptide **1** and analogue **3** in the same environment. Contrarily to LUVs, which are not suitable for measurements using the solution ^1^H NMR method due to their large size leading to a drastic broadening of the signals, isotropic bicelles have come out to be a powerful medium for studying membrane-associated biomolecules. A bicelle is a discoidal lipid aggregate composed of long-chain phospholipid and detergent (often short-chain phospholipid molecules). The long-chain phospholipids are prone to form a bilayer, while the detergent molecules mostly locate within the rim around the bilayer. Bicelles with *q* < 1 (*q* is the molar ratio of long-chain to short-chain lipids) have isotropic tumbling and are ideal for solution NMR studies.^36^^1^H NMR chemical shift assignments of most of the proton signals were effectively accomplished for the peptides according to the Wüthrich procedure^37^ (Supporting Information, Tables S3–S5).

All peptides in the presence of POPG/POCL bicelles exhibited NMR spectral features denoting helical propensity. Upfield shift of the Hα NMR signals (Figure S9), low values of the temperature coefficients of many amide protons (Tables S3–S5), and diagnostic NOEs (Tables S6–S8) suggested that many residues are in a helical conformation. For peptides **1** and **2**, these signatures are observable along the entire sequence, while for peptide **3** they can be observed only for residues following Pro^8^. Moreover, the main difference between peptide **2** and peptide **1** is in the chemical shift values of residues close to the mutation point (i.e., residue 8) with significant upfield shifts observed for the first (Figure S9).

**Figure 9:**
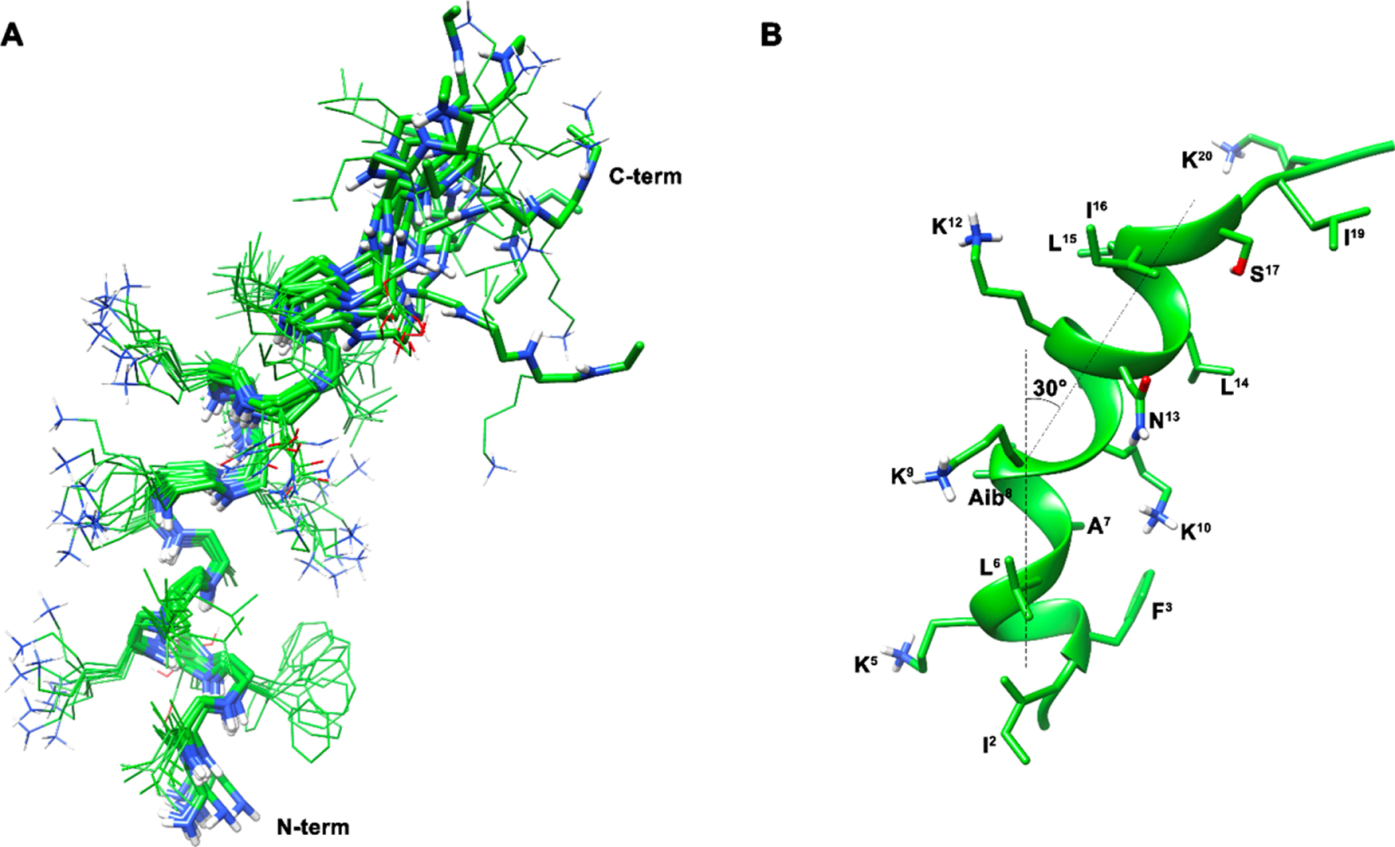
(A) NMR-derived 10 lowest-energy structures of peptide **2**. Heavy atoms are shown in atom-type coloring (carbon, green; nitrogen, blue; oxygen, red; hydrogen, white). For the sake of clarity, only polar hydrogen atoms are shown. (B) Representative structure of peptide **2**. Backbone is shown as a ribbon and helical axes are shown as dotted lines.

For peptide **1**, the presence of many overlapping signals hampered the calculation of a 3D structure, which was possible for peptide **2**. NMR-based structure calculation for peptide **2** gave an ensemble of 10 structures (Figure 9A) satisfying the NMR-derived constraints (violations smaller than 0.10 Å). The backbone is well-defined, apart from the N-terminal and C-terminal residues, with an rmsd of 0.58 Å along residues 2–16. An α-helix from Phe^3^ to Leu^6^ can be observed followed by a 3_10_ helix from Ala^7^ to Lys^9^ and again by an α-helix from Lys^10^ to Ile^16^. The C-terminal tail also has the tendency to form the helix but turns out to be more flexible. As shown in Figure 9B, the structure of peptide **2** can be described as a distorted helix, bent in correspondence to the Aib^8^ residue, due the local formation of a 3_10_ conformation; the bend angle of the NMR structures is about 30°. The observed helix-bend-helix conformation is still amphipathic since the concave side of the peptide anchors many of the hydrophobic residues, while the charged residues Lys^5^, Lys^9^, and Lys^12^ are exposed on the opposite face (Figure 9B). The exception is the Lys^10^ side chain, which is positioned on the hydrophobic face; this can play a role and will be discussed later.

## Discussion

Every year, millions of deaths are caused by infections from bacteria that are resistant to conventional antibiotic treatment and this poses a serious threat to human health on a global scale.^1,38−41^ Although many AMPs are considered lead compounds for the production of novel antibacterial drugs, only a few of them have been approved so far for clinical use^5,42−44^ mainly because of their general cytotoxicity toward mammalian cells and rapid enzymatic degradation, especially in living systems.^45,46^ However, studies aimed at mitigating their cytotoxicity and susceptibility to proteolytic degradation are in progress, including chemical modification of the peptides as well as the exploration of different delivery methods encompassing encapsulation inside vehicles or conjugation to them.^47^

According to the literature, the antimicrobial activity of cationic α-helical peptides against bacteria and fungi, and the cytolytic effect against eukaryotic cells are determined by a large variety of physicochemical features including cationicity, hydrophobicity, and percentage of α-helicity and amphipathicity.^48,49^ One of the biochemical approaches to modulate these properties is the strategic amino acid substitution in the primary structure of AMPs.^50^

In this work, the replacement of Gly^8^ with Aib, Pro, and dPro (peptides **2**–**4**, respectively, Table 1) was aimed at fine-tuning some physicochemical properties such as the α-helical content and the correlated amphipathicity. In particular, the Aib residue is known to stabilize the α-helix or, alternatively, the 3_10_ helix,^24,25^ while the Pro residue induces kinks in transmembrane helices.^26^

As reported in Table 2, the single substitution Gly^8^ → Aib^8^, leading to peptide **2**, conferred the peptide an increased activity against the free-living form of Gram-positive bacterial strains compared to the parent peptide **1** (>8-fold, i.e., 12.5 μM vs ≥100 μM), including multidrug-resistant clinical isolates. In contrast, peptides **3** and **4** were less active than the parent compound. Hence, peptide **2** was further investigated in different biological assays.

Interestingly, peptide **2** showed a higher antibiofilm activity with respect to peptide **1** (Figure 1) leading to a more than 90% reduction of *S*. *aureus* biofilms within 2 h at a concentration of 25 μM. This is an essential feature for the development of new antimicrobial compounds. Indeed, conventional antibiotics (that generally target energy-consuming processes) are usually ineffective when tested against biofilm-associated infections, mainly due to the metabolically inactive state of dormant cells embedded into the extracellular biofilm matrix.^51−53^ Furthermore, long-term antibiotic treatments are required to eradicate biofilms, prolonging antibiotic exposure, which makes bacterial cells more prone to develop resistance.^54−56^

Notably, peptide **2** was also able to induce a significant reduction in CFU within a few minutes at its MIC and 2 x MIC, suggesting a membrane-perturbing mechanism of action, as supported by both the Sytox Green and CF leakage assays (Figures 4 and 7).

Furthermore, stability data in serum revealed that ∼40% of the original amount of peptide **2** remained after 16 h from the addition of bovine serum (Figure 3), highlighting a prolonged half-life compared to most gene-encoded AMPs^57^ including the same parent peptide **1**, the amount of which decreased to 21% after 5 h incubation with fresh human serum.^20^ This is likely due to the presence of the noncoded Aib that should protect the peptides from degradation by plasma proteases,^58^ by preventing their interaction with the serine proteases elastase and thermolysin.^59^ In support of this, Hirano and co-workers designed analogs of the Stripe peptide (21 residues long), carrying different α,α-disubstituted amino acids or side-chain stapling to stabilize their helical structures. Of these, the Aib-containing analogue showed an enhanced antimicrobial activity against *S*. *aureus*; however, it was detectable by HPLC even after 24 h incubation with proteinase K; on the contrary, the parent peptide was totally degraded after 1 h.^60^ While the increase of stability in serum can be a direct consequence of the presence of the nonproteinogenic Aib residue and one of the reasons accounting for the higher activity of peptide **2**, this latter finding deserved further investigations including studies on the peptide aggregation, membrane interaction, and conformational behavior.

First, aggregation studies for peptides **1**–**3** were performed both in aqueous solution and in LUVs mimicking the membrane of Gram-positive bacteria. Peptides **1**–**3** are not aggregated at the concentration tested and are in a monomeric state in solution, as proven by the Nile Red assay. In contrast, a progressive phenomenon of aggregation in LUVs was clearly observed for peptides **1** and **2**, while peptide **3** did not show aggregation at any lipid/peptide ratio.

In addition, the effect of the peptides on the fluidity, charge surface, and mean diameter of liposomes was also measured by dynamic light scattering and the Laurdan assay. After the treatment of LUVs with each peptide, the zeta potential became highly positive and the mean diameter increased. The strong electrostatic interactions established between peptides and LUVs determined positive zeta potential values of +10.9 ± 0.1 mV, + 13 ± 2 mV, and +15 ± 2 mV for peptides **1**, **2**, and **3**, respectively. In the same conditions, an increase of the mean diameter of LUVs (107 ± 1 nm) was recorded in the presence of all peptides. In fact, the mean diameter significantly increased in the presence of peptides **1** and **2**, becoming 175 ± 1 nm and 175 ± 2 nm, respectively, whereas it slightly changed (138 ± 4 nm) in the presence of peptide **3**. Moreover, after treatment of LUVs with the peptides, the membrane fluidity was analyzed, and it was found that the GP parameter changed only for the most active peptide **2** at 20 μM. Specifically, a slight but significant decrease from −0.51 to −0.45 of LUVs occurred just after treatment with peptide **2**, indicating a perturbation of the lipid fluidity in its presence. The peptide/membrane interaction, observed for all peptides, cannot explain their different activities. However, the reorganization of the membrane induced by the peptide, leading to the observed reduction in fluidity probably plays a role in the mechanism of action.

To get further insight into the unique bioactivity of peptide **2**, conformational studies were performed by CD and NMR techniques. CD of peptides **1**–**3** acquired in water and in the presence of LUVs mimicking the Gram-positive bacterial membrane (Figure 8) clearly showed that the peptides are mainly unstructured in water, with peptide **2** being slightly more structured in agreement with the helical stabilization properties of the Aib residue.^24^ In the LUV membrane model, peptides **1**–**3** tend to assume helical structures. It is interesting to note that peptide **2** has the highest helical character in a membrane-mimicking environment (Table 5), in agreement with the design strategy. CD spectra also revealed a clear tendency of peptides **1** and **2** to oligomerize in LUVs, in line with the ThT results.

The conformational behavior of the peptides was also studied by solution NMR using a bicelle system.

The structure of peptide **2** (Figure 9) in POPG/POCL 6:4 bicelles can be described as a bent helix with the point of curvature centered on the Aib residue and formed by an α-helix from Phe^3^ to Leu^6^ followed by a 3_10_ helix from Ala^7^ to Lys^9^ (bend region) and again by an α-helix from Lys^10^ to Ile^16^.

It was not possible to calculate the 3D structure of peptide **1** in bicelle solution to make a direct comparison with its derivative **2**. However, peptide **1** also showed a strong tendency to fold into a helix. In fact, the temperature coefficients of the amide protons and the Hα chemical shifts are very similar for the two peptides, **1** and **2** (Tables S3, S4, and Figure S9) apart around Aib^8^, where both these parameters point to a helix enforcement in peptide **2**. Putting together our NMR and CD data with information from the literature about peptide **1**,^18^ it is conceivable that peptide **1** folds into an α-helix extending itself over all the central residues of the peptide (2–16) with some degree of flexibility around the Gly^8^ residue,^16,18^ which becomes a more rigid bent structure upon the Gly to Aib^8^ mutation passing from **1** to **2**. Concerning peptide **3**, NMR data clearly indicated that the Pro^8^ residue highly destabilizes the helix at its N-terminus while leaving the helical structure at its C-terminus almost unchanged compared to both peptides **1** and **2**. In contrast with the design strategy, the Pro residue did not induce the helix kink but led to the almost complete disappearance of the helix at its N-terminus, probably because the tendency of this region to fold as a helix is not high enough to overcome the helical breaking property of proline.

**Scheme 1:**
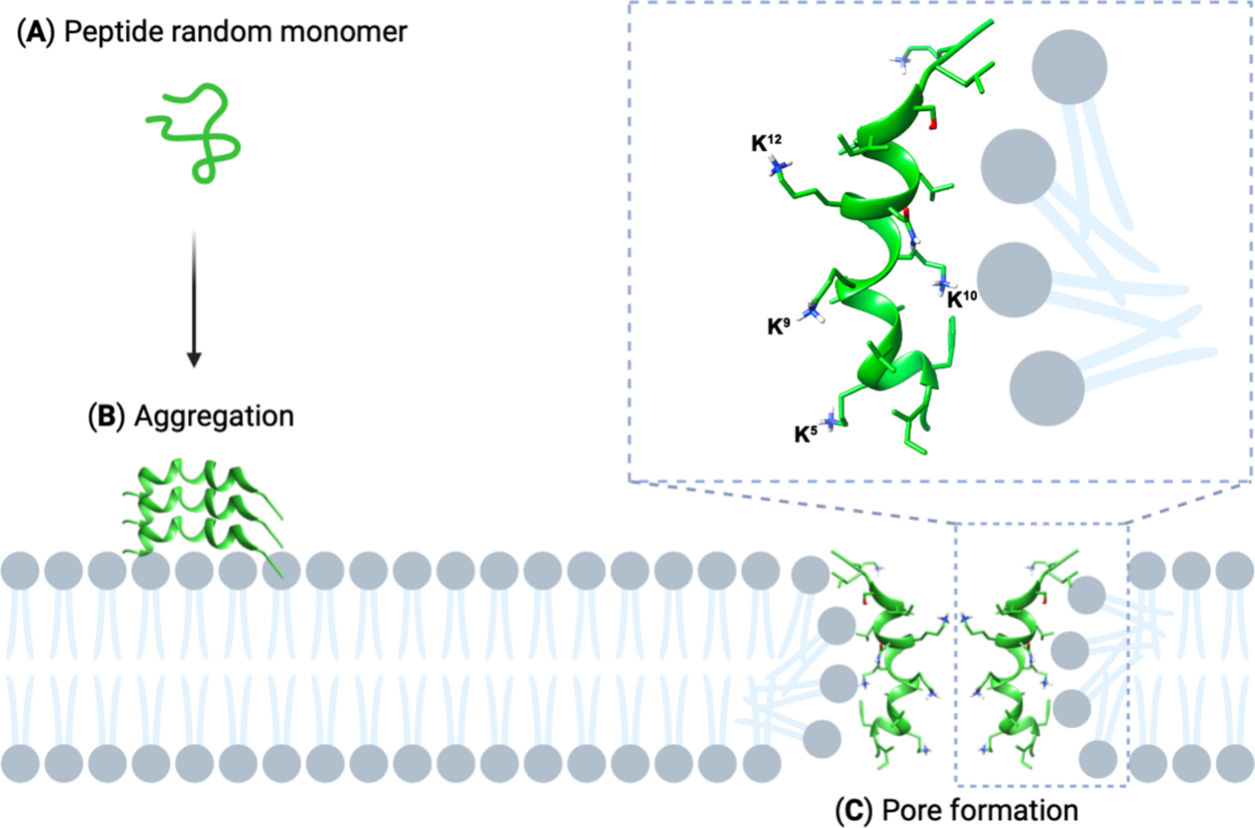
Schematic Description of the Hypothetical Mechanism Involved in Peptide 2 Activity

Taking together all of the obtained information about the peptides with different activity profiles, a plausible mechanism can be hypothesized (Scheme 1).

Esc(1–21) (**1**) and its analogues are monomeric in water where they are largely unstructured (Scheme 1A). Only active peptide **2** showed some tendency to fold in water solution, which could be important for its prominent activity. All peptides interact with the membranes. Within the membrane, peptides **1** and **2** both oligomerize and take a helical conformation (Scheme 1B). The fast membrane perturbation process induced by peptide **2** in the Sytox and leakage assays together with its ability to reduce the membrane fluidity indicate that it can perturb the Gram-positive bacterial membrane probably by the formation of pores, thus exerting its antimicrobial activity. Since the main difference observed between the structures of **1** and **2** was the formation/stabilization of the kink along helixes 2–16 observed for the last peptide, this structural element should play a role in different activities. Notably, the importance of a bent helical structure has long been discussed for other AMPs. Many amphipathic helical AMPs, such as cecropin A, magainin 2, caerin 1.1, maculatin 1.1, and melitin exhibit a kink or bend in their helical folding, typically occurring at the position of a glycine or proline along the amino acid sequence. This particular feature is known to facilitate the interaction between the peptide and the lipid bilayer.^61−63^ It was postulated that the presence of a hinge allows for an optimal alignment between the N- and C-terminal α helices when the peptide interacts with bacterial cell membranes. Moreover, the bent helix can facilitate the formation of pores within the cytoplasmic membrane, by stabilizing toroidal pores.^64^ As a matter of fact, peptide **2** induced a very fast membrane perturbation process (Figure 4). In fact, within the first few minutes from its addition, the highest values of fluorescence intensity were recorded for both *S*. *epidermidis* and *S*. *aureus* bacterial strains. Interestingly, melittin, which has a wide range of bactericidal activity against susceptible and drug-resistant bacteria, like *S*. *aureus*(65) folds as a bent helical structure, likewise to peptide **2**. Interestingly, Vogel and Jahnig found that melittin aggregated in tetramers, forming hydrophilic pores in the membrane.^61^ A similar mechanism of action can be hypothesized also for peptide **1** and even more so for peptide **2**.

The helix-bend-helix conformation of peptide **2** is still amphipathic as the concave side of the peptide anchors many of the hydrophobic residues while the charged Lys residues are positioned on the opposite face (Figure 9B). A notable exception is Lys^10^ positioned within the hydrophobic side of the helix. Since the hydrophobic face should be in contact with the membrane at the interface between hydrophobic and hydrophilic sectors in the postulated toroidal pore (Scheme 1C), the Lys^10^ side chain is perfectly suited to interact with the anionic phospholipid heads, thus helping in pore formation. On the other hand, zwitterionic phospholipids forming the eukaryotic cell membranes would be less efficient at interacting with peptide **2**.

As a matter of fact, peptide **2** did not induce any significant cytotoxic effect up to 50 μM (∼20% reduction of metabolically active cells), a concentration which is 4-fold higher than its MICs against *S*. *aureus* strains. This result differs from that obtained with the analogue bearing Aib residues at three different positions (1, 10, and 18) in the primary structure of Esc(1–21).^22^ These mutations made the last peptide active against Gram-positive bacteria, including *S*. *aureus* but also cytotoxic to human cells at concentrations higher than 4 μM (∼80% reduction of metabolically active cells at 16 μM).

## Conclusions

The current work has highlighted how a single-residue substitution with an Aib residue at a strategic position of peptide **1** increases its activity against both planktonic and sessile forms of Gram-positive bacterial pathogen *S*. *aureus* including MDR strains, improving biostability without resulting in cytotoxicity to mammalian cells at concentrations significantly higher than those displaying antibacterial activity, and thus improving the corresponding therapeutic index. This improvement is at variance with what was previously reported for another analogue of peptide **1** carrying three Aib residues in different positions. It is well known that Aib residues stabilize the helix conformation of a peptide; however, the best activity of peptide **2** could be explained by the combination of different factors, including (i) the higher biostability due to the presence of a nonproteogenic amino acid; (ii) some tendency to fold in water solution; (iii) the higher α helical content in membranes; (iv) the ability to reduce membrane fluidity, and (v) the adoption of a distorted helix bent on Aib^8^, which is probably more suited to facilitate the interaction of the peptide with membrane phospholipids, helping pore formation. Despite the fact that peptides **1** and **2** did not show any significant difference in their aggregation state in solution and/or in a membrane-mimicking environment (LUVs) we cannot exclude, at this stage, discrepancies in their oligomeric state within the peptidoglycan layer surrounding the surface of Gram-positive bacterial cells, likely affecting their translocation through the bacterial cell wall into the target cytoplasmic membrane. Further studies aimed at investigating its *in vivo* antimicrobial efficacy and safety profile are also in progress.

## Methods

### Materials, Microbial Strains, and Cell Line

All *N*^α^-Fmoc-protected conventional amino acids were acquired from GL Biochem Ltd. (Shanghai, China). Fmoc-Aib–OH, Fmoc-dPro-OH, *N*,*N*-diisopropylethylamine (DIEA), piperidine, (1-cyano-2-ethoxy-2 oxoethylidenaminooxy)dimethylamino-morpholino-carbenium hexafluorophosphate (COMU), oxyma, triisopropylsilane (TIS), and trifluoroacetic acid (TFA) were purchased from Iris-Biotech GMBH.

The nondeuterated lipids POPG and POCL and deuterated DHPC-d22 were from Avanti Polar Lipids (Alabaster, AL); CF and MTT were from Merck (St. Quentin Fallavier, France). Sytox Green was purchased from Molecular Probes (Invitrogen, Carlsbad, CA).

For the antimicrobial assays, a panel of Gram-negative bacteria (*Escherichia coli* ATCC 25922; *Acinetobacter baumannii* ATCC 19606; *Pseudomonas aeruginosa* ATCC 27853) and the Gram-positive bacterial strains (*Staphylococcus epidermidis* ATCC 12228; *Staphylococcus aureus* ATCC 25923; *Staphylococcus aureus* MDR 13164462 (#1); *Staphylococcus aureus* MDR 13165968 (#2); *Staphylococcus aureus* MDR 13667073 (#3); *Staphylococcus aureus* MDR 02216108 (#4)) were used in this study. MDR isolates were from the strain collection of Policlinico Umberto I(Sapienza, University of Rome).

For the cytotoxicity assay, HaCaT cells, (AddexBio San Diego, CA, United States) were used.^66,67^ They were grown in Dulbecco’s modified Eagle’s medium supplemented with 4 mM glutamine (DMEMg), 10% heat-inactivated fetal bovine serum (FBS), and 0.1 mg/mL penicillin and streptomycin. The culture was maintained in a humidified incubator at 37 °C and 5% CO_2_.^68^ All other reagents were from Sigma Aldrich (St. Louis, MO, United States).

### Peptide Synthesis

Peptides **1**–**4** were synthesized by the US-SPPS methodology.^69,70^ Each peptide was constructed on the Rink amide resin (loading substitution of 0.72 mmol/g) as the solid support. The Fmoc group was removed from the rink amide linker by using 20% piperidine in dimethylformamide (DMF) solution (0.5 + 1 min) under ultrasound. After washing the resin with DMF (×3) and dichloromethane (DCM, × 3), the first coupling was performed with Fmoc-AA (2 equiv), COMU (2 equiv), oxyma (2 equiv), and DIEA (4 equiv) under ultrasound for 5 min. Then, the peptide elongation was achieved by several cycles of Fmoc deprotections and coupling reactions performed as described above. After the complete elongation, a small amount of the resin was treated with the cleavage cocktail (TFA/TIS/H_2_O, 95:2.5:2.5 *v/v/v*) for 1 h and the identity of each peptide was confirmed through both ESIMS and the ultra high-performance liquid chromatography (UHPLC) analysis. Finally, peptides were removed from the resin along with the protecting groups attached to side-chain amino acids by treatment with the cleavage cocktail. After 3h, each peptide was precipitated in chilly diethyl ether, centrifuged (2 × 15 min, 6000 rpm), and dried in a vacuum. Then, the peptide crudes were dissolved in 10% acetonitrile (MeCN) in H_2_O and purified by RP-HPLC using a Phenomenex Kinetex C18 column (5 μm, 100 Å, 150 × 21.2 mm), with the linear gradient of MeCN (0.1% TFA) in water (0.1% TFA), from 10 to 90% over 30 min, and with a flow rate of 10 mL/min and UV detection at 220 nm.

### Antimicrobial Activity

Antimicrobial susceptibility was evaluated by the broth microdilution assay to determine the MIC as already described by Buommino et al.^71^ The bacterial suspension in Muller Hinton (MH) at a concentration of 2 × 10^6^ cell/mL in the mid-log phase was prepared. Aliquots of 50 μL of the bacterial suspension were added to 50 μL of MH containing serial 2-fold dilutions of the peptides (from 100 to 0.78 μM) that were previously prepared in a 96-well plate. The MIC value was defined as the lowest concentration of the peptide that completely inhibited bacterial growth, after incubation for 16–18 h at 37 °C. Each measurement was performed in triplicate. MICs were obtained from three identical readings of four independent experiments. The antibiofilm activity of the tested peptides was evaluated as reported by Casciaro et al.^72^ Microbial culture was grown at 37 °C until it reached an optical density (OD) of 0.8 (λ = 590 nm), and then it was diluted to a cell density of 1 × 10^6^ colony-forming units (CFUs)/mL.

Aliquots of 100 μL of this suspension were dispensed into the wells of a 96-multiwell plate and incubated for 20 h at 37 °C. After biofilm formation, planktonic cells were removed, and each well was washed twice with 150 μL of PBS to eliminate any nonadherent cells. After washing, each well was filled with PBS supplemented with different 2-fold serial dilutions of peptide **1** and peptide **2** (from 3.12 to 100 μM), and the plate was incubated for 2 h at 37 °C. Each well was washed with PBS twice. Biofilm viability was assessed by adding 150 μL of MTT (0.5 mg/mL) and incubating it for 4 h (at 37 °C). The reaction was then stopped by adding SDS (final concentration equal to 5% v/v). The absorbance of each well was measured at 570 nm with a microplate reader (Infinite M200; Tecan, Salzburg, Austria), and the percentage of biofilm viability was calculated relative to the untreated samples.

### Cytotoxicity

*In vitro* cytotoxicity of peptide **2** was tested using a colorimetric method that relies on the intracellular reduction of the tetrazolium salt MTT. This reduction is carried out by mitochondrial dehydrogenases of metabolically active cells, resulting in the formation of purple formazan crystals.^66^ Briefly, about 4 × 10^4^ HaCaT cells, suspended in DMEM supplemented with 2% FBS, were plated in triplicate wells of a 96-well microtiter plate and incubated overnight at 37 °C and 5% CO_2_. Then, the cells were treated with different concentrations of the peptide (from 50 to 1.56 μM) in a fresh serum-free medium for 24 h. Following the treatment, the medium was removed, and MTT solution in Hank’s buffer (136 mM NaCl, 4.2 mM Na_2_HPO_4_, 4.4 mM KH_2_PO_4_, 5.4 mM KCl, 4.1 mM NaHCO_3_, pH 7.2, supplemented with 20 mM d-glucose) (final concentration 0.5 mg/mL) was added to each well. The plate was then incubated for 4 h at 37 °C and 5% CO_2_. The formazan crystals were dissolved using acidified 2-propanol, and the absorbance of each well was measured at 570 nm using the microplate reader (Infinite M200; Tecan, Salzburg, Austria). Cell viability was expressed as a percentage compared to the control, which consisted of cells without any peptide treatment (100% viability). All data are the mean of three independent experiments ± SEM.

#### Proteolytic Stability

The proteolytic stability of peptide **2** was evaluated in bovine serum (ThermoFisher Scientific, Milan, Italy). Peptide **2** was dissolved in sterile water (200 μM) and incubated with 50% bovine serum at 37 °C. The reaction mixture was incubated at different time intervals (1, 3, 4, 5, 16, and 24 h). An aliquot of the mixture was taken at each pre-established time point and MeCN was added to precipitate serum proteins. Then, the mixture was cooled to 4 °C, centrifuged for 15 min (13,000*g* rpm), and then the supernatant was checked by HPLC using a Phenomenex Jupiter column (4 μm Proteo 90 Å 250 × 21.20 mm) with a linear gradient of MeCN (0.1% TFA) and H_2_O (0.1% TFA) from 10 to 90% in 20 min and with a flow rate of 1 mL/min.^73^ The percentage of the nondegraded peptide was calculated by integrating the peak area of each HPLC chromatogram. Data points are the mean ± SD of three different experiments.

### Membrane Permeabilization Assay

The ability of peptide **2** to alter the bacterial membrane permeability of planktonic cells of *S*. *epidermidis* ATCC 12228 and *S*. *aureus* ATCC 25923 was determined according to Marcellini et al. and Casciaro et al.^15,74^

Approximately 1 × 10^6^ cells in 100 μL of PBS were combined with 1 μM Sytox Green in the dark for 5 min. After peptide addition, the increase in fluorescence intensity, due to the binding of the dye to intracellular nucleic acids, was monitored for 30 min using a microplate reader (Infinite M200, Tecan, with excitation and emission at λ = 485 and 535 nm, respectively) at 37 °C. The peptide concentrations ranged from 0.39 to 12.5 μM. Controls consisted of cells that were not exposed to the peptides. The values correspond to one representative experiment out of three independent experiments.

### Killing Kinetics

The effect of peptide **2** on the viability of *S*. *aureus* ATCC 25923 was evaluated by counting the number of CFUs as previously reported.^75^ Briefly, about 1 × 10^6^ CFU/mL were incubated with peptide **2** at different concentrations (2 × MIC, MIC, 1/2 × MIC, and 1/4 × MIC). Aliquots were withdrawn after 5, 15, 30, 60, 90, and 120 min, appropriately diluted in PBS, and spread onto agar plates for cell viability analysis. The plates were then incubated overnight at 37 °C and the number of CFUs was determined. Controls were samples in the presence of the peptide solvent. The bactericidal activity was determined by comparing the number of viable cells in the control to those treated with the peptides. Data represent the mean ± SD of four independent experiments.

### LUV Preparation

Lipid films of POPG/POCL were prepared by dissolving lipids (POPG/POCL mixture, 6:4, *mol/mol*) in chloroform/methanol (1:1, v/v).^18^ The solvents were evaporated in a rotary vacuum system until a thin film was formed. Complete evaporation of the organic solvent was ensured by applying a rotary vacuum pump for at least 2 h. The lipid film was then hydrated with water for CD experiments or with a CF solution at a self-quenching concentration, i.e., 30 mM (for CF leakage experiments) in 10 mM phosphate buffer containing 40 mM NaCl, 135 mM NaOH, 0.1 mM EDTA, and HCl 6 mM (pH 7.4). After 10 freeze and thaw cycles, the liposome solution was extruded 31 times by two stacked polycarbonate membranes with 100 nm pores, to obtain LUVs. Gel filtration chromatography was used to remove free CF and the final lipid concentration was measured by the Stewart assay.^18^

### Peptide Aggregation

The aggregation of peptides **1**–**3** in aqueous solution was evaluated by using Nile Red (NR) as the fluorophore by calculating the critical aggregation concentration. Each peptide was dissolved in 1,1,1,3,3,3-hexafluoro-2-propanol (HFIP) and different aliquots were taken to prepare the peptide solutions at different concentrations (1, 5, 10, 15, 20, 30, 50, 100, and 200 μM). Then, the organic solvent was evaporated, water (0.5 mL) was added, and each aliquot was sonicated for 15 min and lyophilized. For the experiment, each peptide solution was hydrated with NR solution of 500 nM for 1 h. Each NR spectrum was recorded at a fluorescence emission between 570 and 700 nm (slit width, 5 nm), and an excitation wavelength of 550 nm (slit width, 10 nm). The value of CAC was obtained by fitting the maximum emission fluorescence corresponding wavelength as a function of the peptide concentration using the sigmoidal Boltzmann equation as described previously.^76^ Regarding the peptide aggregation in LUVs made of POPG/POCL (6:4, *mol/mol*), fluorescent probe ThT was used. Each phospholipid (POPG or POCL) was dissolved in chloroform for the preparation of the lipid film at a final concentration of 100 μM. Then, the lipid film, hydrated with 100 mM NaCl, 10 mM Tris-HCl, and 25 μM ThT buffer, pH 7.4, was vortexed for 1 h, and treated to have LUVs as described above.^77^ The peptide aggregation was evaluated by treating LUVs with increasing peptide concentrations of 5, 10, 15, and 20 μM. Each ThT spectrum was recorded by exciting the sample at 450 nm (slit width,10 nm) and recording fluorescence emission at 482 nm (slit width, 5 nm). The tendency of the peptide to aggregate was calculated as *F*–*F*_0_ where *F*_0_ and *F* indicate the value of ThT fluorescence before and after the treatment of LUVs with the peptide, respectively. The exact percentage of aggregation could not be calculated due to the turbidity of the LUV solution after the addition of the peptides at a concentration >20 μM.

### Zeta Potential and Size Measured by Dynamic Light Scattering

LUVs made of POPG/POCL (6:4, *mol/mol*) were prepared at the final concentration of 100 μM as described above. LUVs were incubated with a peptide concentration of 20 μM. Dynamic light scattering (DLS) measurements to calculate the zeta potential and the size of LUVs before and after the peptide treatment were performed using a Zetasizer Nano-ZS (Malvern Instruments, Worcestershire, U.K.).^76^ The analysis was performed with a He–Ne laser 4 mW operating at 633 nm at a scattering angle fixed at 173° and at 25 °C. The results were repeated three times for each sample and each measurement was carried out in triplicate. PDI of the LUVs was also reported.

### Laurdan Assay

The influence of peptides **1**–**3** on the membrane fluidity was explored in the presence of LUVs loaded with Laurdan as the fluorescent probe. Laurdan was encapsulated in the lipid film at a concentration of 0.001 mM and then the lipid film was treated as described above to obtain Laurdan-labeled LUVs.^78^ The perturbation on the membrane fluidity was recorded by treating LUVs with the peptide at a concentration of 20 μM. The spectrum in the absence and presence of peptide was performed by recording the Laurdan emission spectrum from 400 to 550 nm with the excitation wavelength of 365 nm. The perturbation in membrane fluidity was evaluated by calculating the GP parameter as follows: GP = (*I*_440_ – *I*_490_)/(*I*_440_ + *I*_490_), where *I*_440_ and *I*_490_ indicate the fluorescence intensities at the maximum emission wavelength in the ordered and disordered state, respectively.

### CF Leakage Assay

Leakage of CF from POPG/POCL (6:4, *mol*/*mol*) LUVs upon incubation with serial two-fold dilutions of peptide **2** was monitored at 37 °C by the fluorescence increase.^18^ Briefly, CF leakage after peptide addition at different concentrations ranging from 0.39 to 50 μM was monitored for 5 min with a microplate reader (Infinite M200, Tecan, excitation and emission wavelengths were 488 and 520 nm, respectively) at 37 °C. The maximum dye release was obtained after treating LUVs with 0.1% Triton X-100 (final concentration), to completely solubilize the lipid vesicles. The percentage of leakage was calculated according to the equation: leakage (%) = 100(*F*_1_ – *F*_0_)/(*F_t_* – *F*_0_), where *F*_0_ is the initial fluorescence without peptide, and *F*_1_ and *F_t_* are the intensities of the fluorescence achieved upon peptide and Triton X-100 treatment, respectively, at different time points. Data points are the mean ± SD of three different experiments.

### CD Spectroscopy

CD experiments were carried out using a Jasco J-810 spectrometer (Jasco International Co., Ltd. Tokyo, Japan). CD spectra were measured for each peptide in water (20 μM) and in LUVS (POPG/POCL 6:4, *mol/mol*) at a concentration of 500 μM. LUVs were prepared as described above. CD spectra were scanned over a range of 190–260 nm with 1 nm data interval and averaged over 4 scans. Blank sample spectra were subtracted from the raw data, and the CD values were converted to per residue molar ellipticity([θ]) (deg cm^2^ dmol^–1^).

### Sample Preparation for NMR Experiments

For NMR samples, the bicelle solution was composed of POPG/POCL (6:4, *mol/mol*) as long-chain lipids and DHPC short-chain lipid, with *q* = 0.10 and *C*_L_ = 9% (where *q* is the molar ratio of long-chain to short-chain lipids and *C*_L_ is the total w/v phospholipid concentration). The appropriate amounts of stock solutions of the long-chain lipids (POPG/POCL) in chloroform were placed in a glass vial and dried under a stream of nitrogen gas. Bicelles were formed by the stepwise addition of an appropriate amount of DHPC stock solution to buffer and vigorous vortexing after each step. The peptide solution was added to a final concentration of 1 mM and everything was mixed by vortexing. In all NMR samples, 10% D_2_O (v/v) was added for field/frequency locking to a final solution of 250 μL. The pH was checked and adjusted to around 6.5 for each sample.

### NMR Spectroscopy

NMR spectra were recorded on a Bruker Avance NEO 600 MHz spectrometer equipped with a z-gradient 5 mm triple-resonance probe head. All the spectra were recorded at a temperature of 308 K. The spectra were calibrated relative to TSP (0.00 ppm) as an internal standard. One-dimensional (1D) NMR spectra were recorded in Fourier mode with quadrature detection. Two-dimensional (2D) DQF-COSY,^79^ TOCSY,^80^ and NOESY^81^ spectra were recorded in the phase-sensitive mode using the method from States.^82^ Data block sizes were 2048 addresses in *t*_2_ and 512 equidistant *t*_1_ values. A mixing time of 70 ms was used for the TOCSY experiments. NOESY experiments were run with mixing times in the range of 150–300 ms. The water signal was suppressed by gradient echo.^83^ The 2D NMR spectra were processed using the NMRPipe package.^84^ Before Fourier transform, the time domain data matrices were multiplied by shifted sin^2^ functions in both dimensions, and the free induction decay size was doubled in F1 and F2 by zero filling. The qualitative and quantitative analysis of DQF-COSY, TOCSY, and NOESY spectra were achieved using the interactive program package XEASY.^85^^3^*J*_HN-Hα_ couplings were difficult to measure because of broad lines. The temperature coefficients of the amide proton chemical shifts were calculated from 1D ^1^H NMR and 2D TOCSY experiments performed at different temperatures in the range 298–313 K by means of linear regression.

The NOE-based distance restraints were obtained from the NOESY spectra of peptide **2** collected with a mixing time of 100 ms. The NOE cross peaks were integrated with the XEASY program and were converted into upper distance bounds using the CALIBA program incorporated into the program package CYANA.^86^ Only the NOE-derived constraints were considered in the annealing procedures. The restraints applied during the calculations are reported in Table S7. An ensemble of 200 structures was generated with the simulated annealing of the program CYANA. Then, 10 structures were chosen, whose interproton distances best fitted NOE-derived distances, and refined through successive steps of restrained and unrestrained energy minimization calculations using the Discover algorithm (Accelrys, San Diego, CA) and the consistent valence force field.^87^ The minimization decreased the total energy of the structures; no residue was found in the disallowed region of the Ramachandran plot. The final structures were analyzed by using the InsightII program (Accelrys, San Diego, CA). Molecular graphics images were realized using the UCSF Chimera package.^88^

## Data Availability

Data reported in this work are available upon request to corresponding authors.

## Supporting Information Available

The following is the Supporting Information related to this article: Analytical data of peptides **1**–**4**; resistance profile of multidrug-resistant *S*. *aureus* strains; NMR data of peptides **1**–**3**; NMR-based restraints used in the structure calculation of peptide **2**; HPLC chromatograms of peptides **1**–**4**; and chemical shift deviations of peptides **1**–**3** (PDF)

## Notes

**Statistical Analysis** All data are presented as means ± SD or SEM GraphPad Prism 8.0 software (San Diego, CA) was used for analysis.
