# Supplementary material for: Strategic Single-Residue Substitution in the Antimicrobial Peptide Esc(1–21) Confers Activity against Staphylococcus aureus, Including Drug-Resistant and Biofilm Phenotype
Source: ACS Infect Dis. 2024 Jun 7;10(7):2403–18. doi: 10.1021/acsinfecdis.4c00130 (PMC11250030; doi:10.1021/acsinfecdis.4c00130)
Supplement: Supplementary file 1 — id4c00130_si_001.pdf [file id4c00130_si_001.pdf]

Supporting Information

A strategic single-residue substitution in the antimicrobial peptide Esc(1-21) confers activity against *Staphylococcus aureus*, including drug-resistant and biofilm phenotype

Maria Rosa Loffredo[a+], Bruno Casciaro[a+], Rosa Bellavita[b+], Cassandra Troiano[c], Diego Brancaccio[b], Floriana Cappiello[a], Francesco Merlino[b], Stefania Galdiero[b], Giancarlo Fabrizi[d], Paolo Grieco[b], Lorenzo Stella[c], Alfonso Carotenuto[b*], Maria Luisa Mangoni[a*]

[a] Department of Biochemical Sciences, Laboratory Affiliated to Istituto Pasteur Italia-Fondazione Cenci Bolognetti, Sapienza University of Rome, 00185 Rome, Italy;

[b] Department of Pharmacy, University of Naples "Federico II", 80131 Naples, Italy

[c] Department of Chemical Science and Technologies, University of Rome Tor Vergata, 00133 Rome, Italy

[d] Department of Chemistry and Technology of Drugs, "Department of Excellence 2018–2022", Sapienza University of Rome, 00185 Rome, Italy;

* *Correspondence to:* Alfonso Carotenuto (alfocaro@unina.it) and Maria Luisa Mangoni (marialuisa.mangoni@uniroma1.it)

Table S1. ESI-MS of synthesized peptides.

Peptide	Calculated mass	Found mass
**1**	[M+2H] $]^{+}$/2=1093.3	[M+2H]$^{+}$/2==1093.2
	[M+3H] $]^{+}$/3=729.2	[M+3H] $]^{+}$/3=729.5
**2**	[M+2H]$^{+}$/2=1107.4	[M+2H]$^{+}$/2=1107.3
	[M+3H]$^{+}$/3=738.6	[M+3H]$^{3+}$/3=738.9
**3**	[M+2H] $]^{+}$/2=1113.4	[M+2H] $]^{+}$/2=1113.2
	[M+3H] $]^{+}$/3=742.6	[M+3H] $]^{+}$/3=742.9
**4**	[M+2H] $]^{+}$/2=1113.4	[M+2H] $]^{+}$/2=1113.2
	[M+3H] $]^{+}$/3=742.6	[M+3H] $]^{+}$/3=742.9

Table S2. Resistance profile multi-drug resistant *S. aureus* strains

**Strain**	**Resistance profile**
S. aureus #1	BEN – CLI – ERI – GEN – LEV – OXA
S. aureus #2	BEN – CLI – DAP – ERI – LEV – OXA -VAN
S. aureus #3	AZI – BEN – CIP – CLI – ERI – LEV – MOX – OXA
S. aureus #4	BEN – CIP – LEV – MOX – OXA

AZI, azithromycin; BEN, benzylpenicillin; CIP, ciprofloxacin; CLI, clindamycin; DAP, daptomycin; ERI, erythromycin; GEN, gentamycin; LEV, levofloxacin; MET, methicillin; MOX, moxifloxacin; OXA, oxacillin; VAN, vancomycin;

Table S3. ^{1}H NMR resonance assignments[a] of peptide **1** in bicelle solution

residue	NH (-Δδ/ΔT)[b]	C$^{\alpha}$H	C$^{\beta}$H	Others
Gly 1		3.94,3.97		
Ile 2	8.94 (3.0)	3.97	1.78	1.33, 1.13, 0.71(γ); 0.92(δ)
Phe 3	8.57 (3.8)	4.47	3.17, 3.10	7.31(δ); 7.22(ε); 7.10(ζ)
Ser 4	8.44 (6.1)	4.19	3.98	
Lys 5	8.10 (7.1)	3.94	1.92, 1.80	1.47(γ); 1.71(δ); 2.98(ε)
Leu 6	8.14 (4.8)	4.22	1.70	1.58(γ); 0.90(δ)
Ala 7	8.14 (5.1)	4.13	1.35	
Gly 8	8.34 (3.8)	3.77, 4.02		
Lys 9	7.99 (1.0)	4.13	1.95	1.50(γ); 1.72(δ)
Lys 10	7.97 (2.3)	4.24	1.92	1.49(γ); 1.74(δ); 3.01(ε)
Ile 11	8.26 (5.0)	3.81	1.97	1.72, 1.18. 0.93(γ); 0.85(δ)
Lys 12	8.28 (3.9)	3.90	1.94	1.51 (γ); 1.72(δ); 2.97(ε)
Asn 13	7.98 (3.4)	4.52	2.93, 2.88	7.65, 6.90(δ)
Leu 14	8.05 (3.6)	4.20	1.82	1.62(γ); 0.93(δ)
Leu 15	8.09 (4.3)	4.13	1.91	1.56(γ); 0.85(δ)
Ile 16	8.09 (4.4)	3.90	2.00	1.72, 1.25, 0.97(γ); 0.90(δ)
Ser 17	8.09 (3.3)	4.31	3.97	
Gly 18	8.20 (2.9)	3.85		
Leu 19	7.98 (2.6)	4.31	1.83	1.63(γ); 0.90(δ)
Lys 20	7.98 (2.3)	4.26	1.90	1.48(γ); 1.72(δ); 2.99(ε)
Gly 21	8.16 (3.4)	3.93		

[a] Obtained at 35°C, pH = 6.5, with TSP (δ 0.00 ppm) as reference shift. Chemical shifts are accurate to ±0.02 ppm. -Δδ/ΔT = temperature coefficients (ppb/K). $CONH_2$ (C-terminal amide protons): 7.11, 7.43 ppm

Table S4. ^{1}H NMR resonance assignments[a] of peptide **2** in bicelle solution

residue	NH (-Δδ/ΔT)[b]	C$^{\alpha}$H	C$^{\beta}$H	Others
Gly 1		4.10, 3.97		
Ile 2	8.94 (3.2)	3.94	1.75	1.35, 1.13, 0.70(γ); 0.81(δ)
Phe 3	8.59 (4.2)	4.43	3.19, 3.10	7.31(δ); 7.19(ε); 7.17(ζ)
Ser 4	8.57 (6.2)	4.15	3.95	
Lys 5	8.06 (5.2)	3.94	1.92, 1.80	1.47(γ); 1.71(δ); 2.98(ε)
Leu 6	7.92 (4.2)	4.16	1.94	1.54(γ); 0.90(δ)
Ala 7	8.20 (4.9)	3.95	1.34	
Aib 8	8.29 (4.5)		1.58, 1.41	
Lys 9	7.70 (0.2)	3.95	1.95	1.50(γ); 1.72(δ)
Lys 10	7.97 (2.1)	4.18	1.92	1.49(γ); 1.74(δ); 3.01(ε)
Ile 11	8.24 (4.3)	3.73	2.04	1.68, 1.18, 0.87(γ); 0.79(δ)
Lys 12	8.24 (3.6)	3.83	1.80	1.43 (γ); 1.69(δ); 2.97(ε)
Asn 13	8.11 (4.8)	4.49	2.95, 2.86	7.65, 6.88(δ)
Leu 14	8.03 (3.5)	4.17	1.94	1.62(γ); 0.92(δ)
Leu 15	8.11 (5.1)	4.09	1.95	1.54(γ); 0.84(δ)
Ile 16	8.14 (3.7)	3.84	2.01	1.72, 1.25, 0.96(γ); 0.90(δ)
Ser 17	8.09 (3.6)	4.28	3.94	
Gly 18	8.18 (3.8)	3.82		
Leu 19	7.97 (3.9)	4.30	1.84	1.61(γ); 0,89(δ)
Lys 20	7.95 (3.4)	4.26	1.90	1.52(γ); 1.68(δ); 3.01(ε)
Gly 21	8.13 (4.1)	3.93		

[a] Obtained at 35°C, pH = 6.5, with TSP (δ 0.00 ppm) as reference shift. Chemical shifts are accurate to ±0.02 ppm. -Δδ/ΔT = temperature coefficients (ppb/K). $CONH_2$ (C-terminal amide protons): 7.11, 7.42 ppm.

Table S5. ^{1}H NMR resonance assignments[a] of peptide **3** in bicelle solution

residue	NH (-Δδ/ΔT)[b]	$C^{\alpha}H$	$C^{\beta}H$	Others
Gly 1		4.09, 3.94		
Ile 2	8.95 (3.4)	3.97	1.73	1.21, 1.17, 0.63(γ); 0.76(δ)
Phe 3	8.52 (5.6)	4.54	3.12, 3.30	7.34(δ); 7.22(ε); 7.17(ζ)
Ser 4	8.08 (1.7)	4.29	4.00	
Lys 5	8.04 (8.6)	4.28	1.85, 2.00	1.50(γ); 1.70(δ); 3.01(ε)
Leu 6	7.69 (4.3)	4.33	1.75	1.61(γ); 0.86(δ)
Ala 7	7.68 (6.0)	4.54	1.38	
Pro 8		4.30	2.17, 2.33	2.01(γ); 3.91, 3.76 (δ)
Lys 9	8.55 (5.8)	4.04	1.88	1.54(γ); 1.72(δ); 3.01(ε)
Lys 10	7.99 (0.1)	4.23	1.92	1.48(γ); 1.73(δ); 3.00(ε)
Ile 11	7.88 (4.9)	3.85	1.96	1.21, 0.91(γ); 0.86(δ)
Lys 12	8.23 (5.9)	3.87	1.89	1.45 (γ); 1.71(δ); 2.97(ε)
Asn 13	7.98 (4.1)	4.51	2.87, 2.88	7.63, 6.88(δ)
Leu 14	7.93 (3.9)	4.19	1.81	1.62(γ); 0.92(δ)
Leu 15	8.00 (5.5)	4.13	1.83	1.56(γ); 0.85(δ)
Ile 16	8.02 (4.8)	3.91	1.96	1.24, 0.96(γ); 0.89(δ)
Ser 17	8.09 (4.1)	4.31	3.95	
Gly 18	8.19 (4.0)	4.01, 3.84		
Leu 19	7.97 (4.2)	4.31	1.75	1.61(γ); 0.89(δ)
Lys 20	7.99 (3.8)	4.29	1.87	1.48(γ); 1.71(δ); 3.00(ε)
Gly 21	8.15 (5.0)	3.93		

[a] Obtained at 35°C, pH = 6.5, with TSP (δ 0.00 ppm) as reference shift. Chemical shifts are accurate to ±0.02 ppm. -Δδ/ΔT = temperature coefficients (ppb/K). $CONH_2$ (C-terminal amide protons): 7.10, 7.43 ppm.

Table S6. NOE derived uppper limit constraits for peptide **1**

2	ILE	HN	2	ILE	QG2	6.53
2	ILE	HN	2	ILE	HG12	5.50
2	ILE	HN	2	ILE	HG13	5.50
2	ILE	HN	2	ILE	QD1	6.53
2	ILE	HN	3	PHE	HN	5.50
2	ILE	HA	2	ILE	QD1	5.23
2	ILE	HB	3	PHE	HN	3.73
2	ILE	QG2	3	PHE	HN	5.07
2	ILE	QG1	3	PHE	HN	5.34
3	PHE	HN	3	PHE	QB	3.32
3	PHE	HA	3	PHE	QD	6.44
3	PHE	HA	6	LEU	HN	4.35
3	PHE	QB	4	SER	HN	4.18
4	SER	HN	5	LYS+	HN	4.07
4	SER	HA	5	LYS+	HN	3.21
5	LYS+	HN	5	LYS+	HB2	3.30
5	LYS+	HN	5	LYS+	HB3	3.30
5	LYS+	HN	5	LYS+	QG	6.19
5	LYS+	HN	5	LYS+	QD	5.14
5	LYS+	HN	5	LYS+	QE	6.38
5	LYS+	HA	5	LYS+	QD	6.38
5	LYS+	QB	5	LYS+	QE	6.01
6	LEU	HN	6	LEU	HG	3.76
6	LEU	HN	6	LEU	QQD	6.45
6	LEU	HA	6	LEU	QQD	5.25
7	ALA	HN	7	ALA	QB	4.14
7	ALA	HN	8	GLY	HN	3.45
7	ALA	HA	8	GLY	HN	3.33
7	ALA	QB	8	GLY	HN	4.76
8	GLY	HN	9	LYS+	HN	3.55
9	LYS+	HN	9	LYS+	HA	2.86
9	LYS+	HN	9	LYS+	QB	3.77
9	LYS+	HN	9	LYS+	QG	5.04
9	LYS+	HN	9	LYS+	QD	6.38
10	LYS+	HN	10	LYS+	HA	2.93
10	LYS+	HN	10	LYS+	QB	3.87
10	LYS+	HN	10	LYS+	QG	5.54
10	LYS+	HN	10	LYS+	QD	5.69
10	LYS+	HN	10	LYS+	QE	6.38
10	LYS+	HN	11	ILE	HN	3.27
10	LYS+	HA	10	LYS+	QD	5.20
10	LYS+	HA	10	LYS+	QE	6.38
10	LYS+	HA	11	ILE	HN	3.30
10	LYS+	QB	10	LYS+	QE	6.94
11	ILE	HN	11	ILE	HB	2.71
11	ILE	HN	11	ILE	QG2	4.39
11	ILE	HN	11	ILE	QG1	3.38
11	ILE	HN	11	ILE	QD1	4.98
11	ILE	HA	11	ILE	QG2	3.68
11	ILE	HA	11	ILE	HG12	3.95
11	ILE	HA	11	ILE	HG13	3.95
11	ILE	HA	11	ILE	QD1	4.48
11	ILE	HA	14	LEU	HN	3.48
11	ILE	HB	11	ILE	QD1	3.64
12	LYS+	HN	12	LYS+	QB	3.90

12	LYS+	HN	12	LYS+	QG	5.73
12	LYS+	HN	12	LYS+	QD	5.94
12	LYS+	HN	13	ASN	HN	3.33
12	LYS+	HA	12	LYS+	QE	6.38
12	LYS+	HA	13	ASN	HN	3.21
13	ASN	HN	13	ASN	HB2	3.14
13	ASN	HN	13	ASN	HB3	3.14
13	ASN	HA	16	ILE	HB	3.45
13	ASN	HB2	14	LEU	HN	3.67
13	ASN	HB3	14	LEU	HN	3.67
13	ASN	QB	14	LEU	HN	3.14
14	LEU	HN	14	LEU	HG	4.04
14	LEU	HN	14	LEU	QQD	5.93
14	LEU	HA	14	LEU	QQD	5.06
15	LEU	HN	15	LEU	HA	2.90
15	LEU	HN	15	LEU	QB	3.74
15	LEU	HN	15	LEU	HG	3.17
15	LEU	HN	15	LEU	QQD	6.49
15	LEU	HA	15	LEU	HG	3.14
15	LEU	HA	15	LEU	QQD	5.34
16	ILE	HN	16	ILE	HA	2.83
16	ILE	HN	16	ILE	HB	2.80
16	ILE	HN	16	ILE	QG2	4.51
16	ILE	HN	16	ILE	QG1	3.33
16	ILE	HN	16	ILE	QD1	3.95
16	ILE	HA	16	ILE	HB	2.93
16	ILE	HA	16	ILE	QG2	4.05
16	ILE	HA	16	ILE	QG1	3.04
16	ILE	HA	16	ILE	QD1	3.77
16	ILE	HB	16	ILE	QD1	3.71
16	ILE	QG2	16	ILE	HG12	4.14
16	ILE	QG2	16	ILE	HG13	4.14
17	SER	HN	17	SER	HA	2.83
17	SER	HA	18	GLY	HN	3.61
18	GLY	HN	19	LEU	HN	3.11
19	LEU	HN	19	LEU	HA	2.80
19	LEU	HN	19	LEU	QB	3.65
19	LEU	HN	19	LEU	HG	3.17
19	LEU	HN	19	LEU	QQD	5.40
19	LEU	HA	19	LEU	HG	3.45
19	LEU	HA	19	LEU	QQD	5.03
20	LYS+	HN	20	LYS+	QG	6.38
20	LYS+	HN	20	LYS+	QD	3.56
20	LYS+	HN	20	LYS+	QE	6.38
20	LYS+	HN	21	GLY	HN	3.05
20	LYS+	HA	20	LYS+	QD	5.20
20	LYS+	HA	20	LYS+	QE	6.38
20	LYS+	HA	21	GLY	HN	3.39
20	LYS+	QB	20	LYS+	QE	7.25
21	GLY	HN	22	CNH2	HN1	5.22

Table S7. NOE derived uppper limit constraits for peptide **2**

1	GLY	QA	4	SER	HN	4.33
2	ILE	HN	2	ILE	HB	3.30
2	ILE	HN	2	ILE	QG2	5.10
2	ILE	HN	2	ILE	HG12	4.69
2	ILE	HN	2	ILE	HG13	4.69
2	ILE	HN	2	ILE	QD1	6.31
2	ILE	HN	3	PHE	HN	3.70
2	ILE	HA	2	ILE	HB	3.02
2	ILE	HA	2	ILE	QD1	5.01
2	ILE	HA	5	LYS+	HN	3.41
2	ILE	HB	3	PHE	HN	3.64
2	ILE	QG2	3	PHE	HN	5.07
2	ILE	QG1	3	PHE	HN	5.00
3	PHE	HN	3	PHE	QB	3.41
3	PHE	HA	3	PHE	QD	6.63
3	PHE	HA	4	SER	HN	3.64
3	PHE	HA	6	LEU	HN	3.40
3	PHE	QB	4	SER	HN	3.88
4	SER	HN	5	LYS+	HN	3.61
4	SER	HN	6	LEU	HN	4.66
4	SER	HA	5	LYS+	HN	3.05
4	SER	HA	7	ALA	HN	3.42
5	LYS+	HN	5	LYS+	HB2	3.58
5	LYS+	HN	5	LYS+	HB3	3.58
5	LYS+	HN	5	LYS+	QB	2.78
6	LEU	HN	6	LEU	HG	3.89
6	LEU	HN	6	LEU	QQD	6.67
6	LEU	HA	6	LEU	QQD	6.05
6	LEU	HA	8	AIB	HN	3.55
6	LEU	HA	9	LYS+	HN	3.70
7	ALA	HN	7	ALA	QB	4.11
7	ALA	HA	8	AIB	HN	3.33
7	ALA	QB	8	AIB	HN	4.61
8	AIB	HN	8	AIB	QB2	3.89
8	AIB	HN	9	LYS+	HN	3.45
8	AIB	QB2	9	LYS+	HN	3.50
9	LYS+	HN	9	LYS+	QB	4.08
9	LYS+	HN	10	LYS+	HN	3.33
9	LYS+	HA	10	LYS+	HN	3.33
9	LYS+	HA	12	LYS+	HN	3.39
10	LYS+	HN	10	LYS+	QB	4.05
10	LYS+	HN	11	ILE	HN	3.17
10	LYS+	HA	11	ILE	HN	3.52
10	LYS+	HA	13	ASN	HN	3.14
11	ILE	HN	11	ILE	HB	2.71
11	ILE	HN	11	ILE	QG2	4.54
11	ILE	HN	11	ILE	QG1	4.22
11	ILE	HN	11	ILE	QD1	5.38
11	ILE	HA	11	ILE	QG2	4.17
11	ILE	HA	11	ILE	QG1	3.70
11	ILE	HA	11	ILE	QD1	4.98
11	ILE	HA	14	LEU	HN	3.46
11	ILE	HA	14	LEU	QQD	6.39
12	LYS+	HA	15	LEU	HN	3.39
13	ASN	HN	13	ASN	HB2	3.21

13	ASN	HN	13	ASN	HB3	3.21
13	ASN	HN	13	ASN	QB	2.80
13	ASN	HA	16	ILE	HB	3.36
13	ASN	HB2	14	LEU	HN	3.64
13	ASN	HB3	14	LEU	HN	3.64
14	LEU	HN	14	LEU	QB	4.08
14	LEU	HN	14	LEU	HG	4.07
14	LEU	HN	14	LEU	QQD	6.58
14	LEU	HA	14	LEU	QQD	5.12
14	LEU	HA	17	SER	HN	4.14
15	LEU	HN	15	LEU	QB	3.83
15	LEU	HN	15	LEU	HG	3.39
15	LEU	HN	15	LEU	QQD	6.39
15	LEU	HA	15	LEU	HG	3.61
15	LEU	HA	15	LEU	QQD	5.49
16	ILE	HN	16	ILE	HA	2.86
16	ILE	HN	16	ILE	HB	2.83
16	ILE	HN	16	ILE	QG2	4.82
16	ILE	HN	16	ILE	QG1	3.63
16	ILE	HN	16	ILE	QD1	4.67
16	ILE	HA	16	ILE	HB	2.80
16	ILE	HA	16	ILE	QG2	4.14
16	ILE	HA	16	ILE	QG1	3.40
16	ILE	HA	16	ILE	QD1	4.14
16	ILE	HB	16	ILE	QD1	4.05
17	SER	HN	17	SER	QB	4.14
18	GLY	HN	19	LEU	HN	2.96
19	LEU	HN	19	LEU	HA	2.74
19	LEU	HN	19	LEU	QB	3.74
19	LEU	HN	19	LEU	HG	3.21
19	LEU	HN	19	LEU	QQD	6.05
19	LEU	HA	19	LEU	HG	3.58
19	LEU	HA	19	LEU	QQD	5.15
20	LYS+	HN	20	LYS+	HA	2.83
20	LYS+	HN	20	LYS+	QB	4.11
20	LYS+	HN	21	GLY	HN	2.83
20	LYS+	HA	21	GLY	HN	3.24
21	GLY	HN	22	CNH2	HN1	4.60

Table S8. NOE derived uppper limit constraits for peptide **3**

2	ILE	HN	2	ILE	QG2	6.53
2	ILE	HN	2	ILE	QG1	5.34
2	ILE	HN	2	ILE	QD1	6.53
2	ILE	HN	3	PHE	HN	4.72
2	ILE	HA	2	ILE	QD1	6.53
2	ILE	HB	3	PHE	HN	4.66
2	ILE	QG2	3	PHE	HN	6.53
2	ILE	QG2	3	PHE	QD	8.65
2	ILE	QG2	3	PHE	QE	8.65
3	PHE	HN	3	PHE	QB	3.59
3	PHE	HN	4	SER	HN	4.01
3	PHE	HA	3	PHE	QD	7.62
3	PHE	HB2	4	SER	HN	4.91
3	PHE	HB3	4	SER	HN	4.91
4	SER	QB	5	LYS+	HN	5.54
5	LYS+	HN	5	LYS+	QB	3.51
5	LYS+	HN	6	LEU	HN	3.92
5	LYS+	HA	5	LYS+	QD	6.38
6	LEU	HN	6	LEU	HG	5.34
6	LEU	HN	6	LEU	QQD	7.63
6	LEU	HA	6	LEU	QQD	6.64
7	ALA	HN	8	PRO	QD	5.34
8	PRO	HA	11	ILE	HN	3.45
9	LYS+	HN	10	LYS+	HN	4.26
9	LYS+	HA	12	LYS+	HN	3.51
11	ILE	HN	11	ILE	HB	3.79
11	ILE	HN	11	ILE	QG1	6.38
11	ILE	HN	11	ILE	QD1	6.53
11	ILE	HN	12	LYS+	HN	4.01
11	ILE	HA	11	ILE	QD1	5.78
11	ILE	HA	14	LEU	HN	3.47
12	LYS+	HN	13	ASN	HN	3.89
13	ASN	HN	13	ASN	QB	3.32
13	ASN	HA	16	ILE	HN	3.56
13	ASN	QB	14	LEU	HN	3.91
14	LEU	HN	14	LEU	HG	5.50
14	LEU	HN	14	LEU	QQD	7.63
14	LEU	HA	14	LEU	QQD	6.95
14	LEU	HA	17	SER	HN	3.57
15	LEU	HN	15	LEU	HG	4.72
15	LEU	HN	15	LEU	QQD	7.63
15	LEU	HA	15	LEU	QQD	6.98
15	LEU	HA	18	GLY	HN	3.69
16	ILE	HN	16	ILE	HB	3.86
16	ILE	HN	16	ILE	QG1	5.79
16	ILE	HN	16	ILE	QD1	6.47
16	ILE	HN	17	SER	HN	5.50
16	ILE	HA	16	ILE	QD1	5.91
16	ILE	QD1	17	SER	HN	6.53
18	GLY	HN	19	LEU	HN	3.83
19	LEU	HN	19	LEU	HG	5.28
19	LEU	HN	19	LEU	QQD	7.63
19	LEU	HN	20	LYS+	HN	5.50
19	LEU	HA	19	LEU	QQD	6.58
20	LYS+	HN	21	GLY	HN	3.89

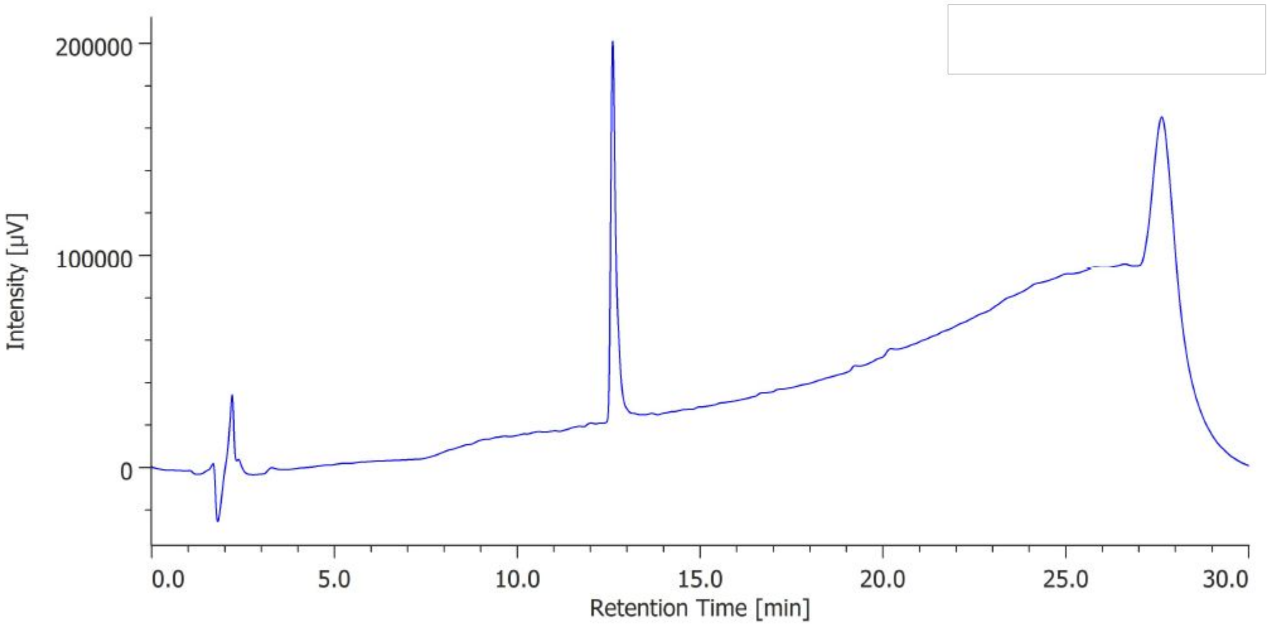

Figure S1. Chromatogram of Esc(1-21) (peptide **1**) obtained by an analytical HPLC (Jasco LC-NetII/ADC) equipped with a Phenomenex Jupiter 4u Proteo column, 90 Å, 150 mm × 4.6 mm, and monitored by UV detection at 220 nm [linear gradient 10-90% MeCN (0.1% TFA) in H_2O (0.1% TFA) over 20 min, flow rate of 1 mL/min].

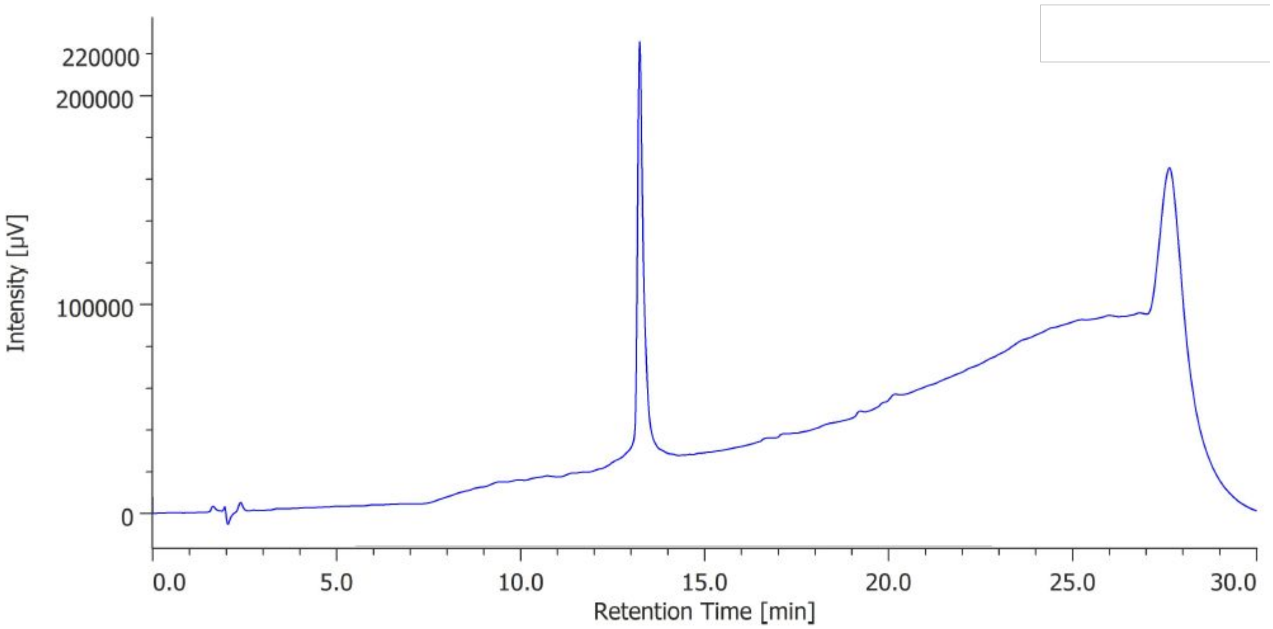

Figure S2. Chromatogram of [Aib^8]-Esc(1-21) (peptide **2**) obtained by an analytical HPLC (Jasco LC-NetII/ADC) equipped with a Phenomenex Jupiter 4u Proteo column, 90 Å, 150 mm × 4.6 mm, and monitored by UV detection at 220 nm [linear gradient 10-90% MeCN (0.1% TFA) in H_2O (0.1% TFA) over 20 min, flow rate of 1 mL/min].

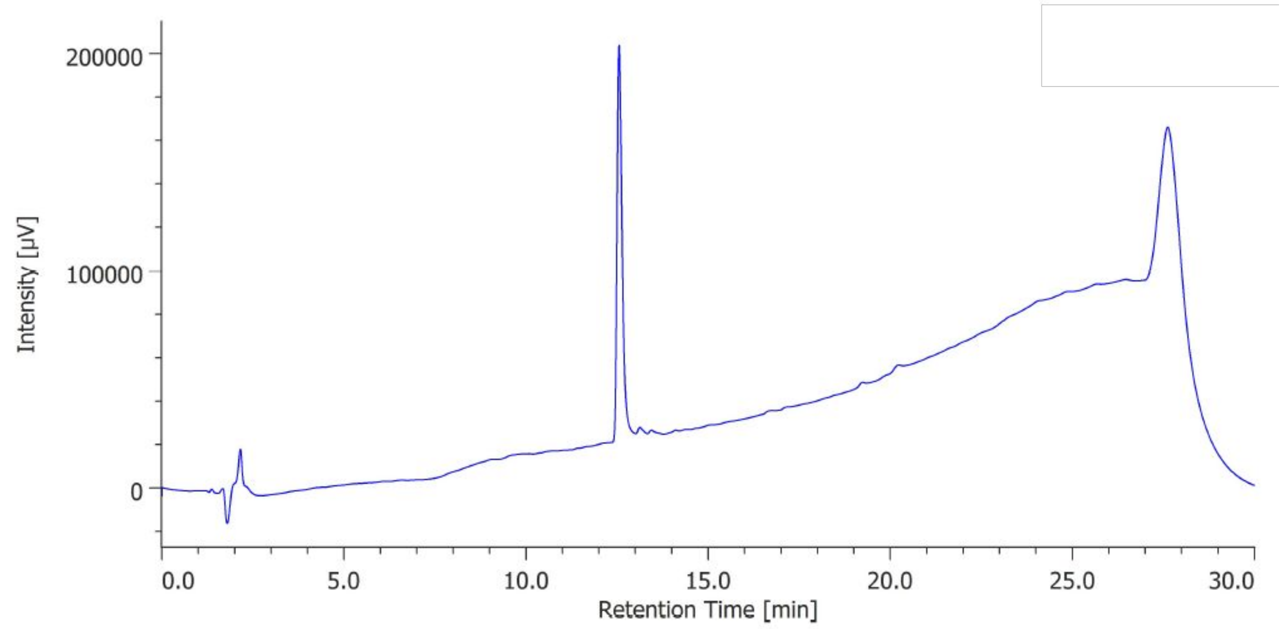

Figure S3. Chromatogram of [Pro^8]-Esc(1-21) (peptide **3**) obtained by an analytical HPLC (Jasco LC-NetII/ADC) equipped with a Phenomenex Jupiter 4u Proteo column, 90 Å, 150 mm × 4.6 mm, and monitored by UV detection at 220 nm [linear gradient 10-90% MeCN (0.1% TFA) in H_2O (0.1% TFA) over 20 min, flow rate of 1 mL/min].

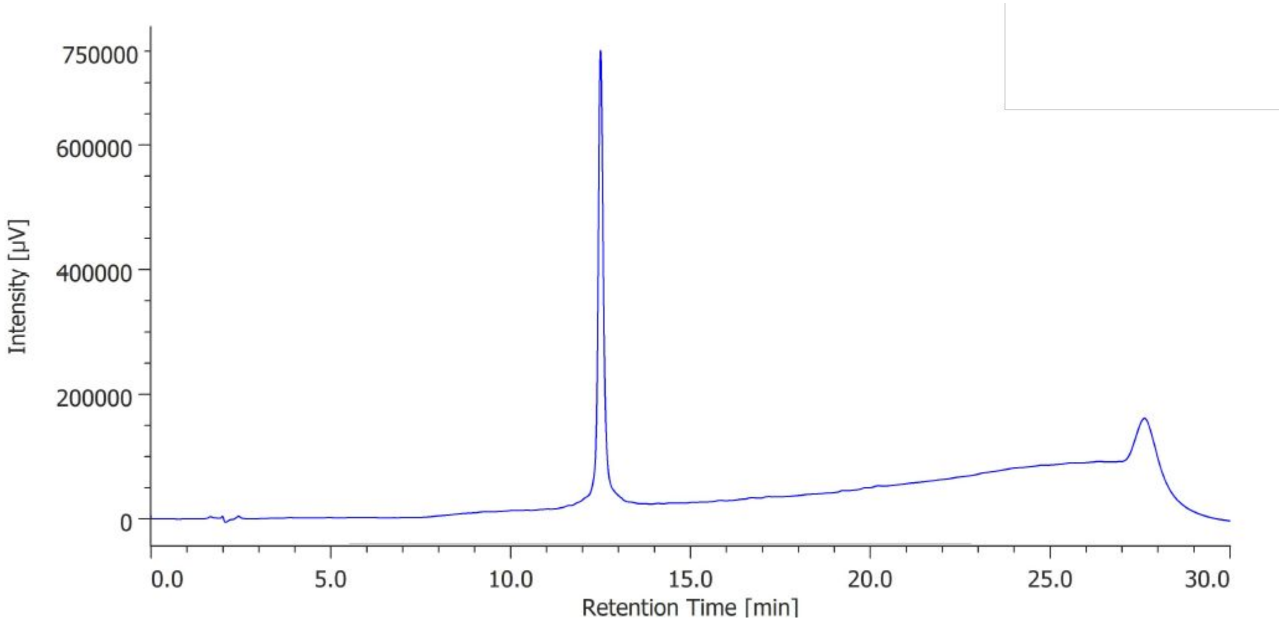

Figure S4. Chromatogram of [DPro^8]-Esc(1-21) (peptide **4**) obtained by an analytical HPLC (Jasco LC-NetII/ADC) equipped with a Phenomenex Jupiter 4u Proteo column, 90 Å, 150 mm × 4.6 mm, and monitored by UV detection at 220 nm [linear gradient 10-90% MeCN (0.1% TFA) in H_2O (0.1% TFA) over 20 min, flow rate of 1 mL/min].

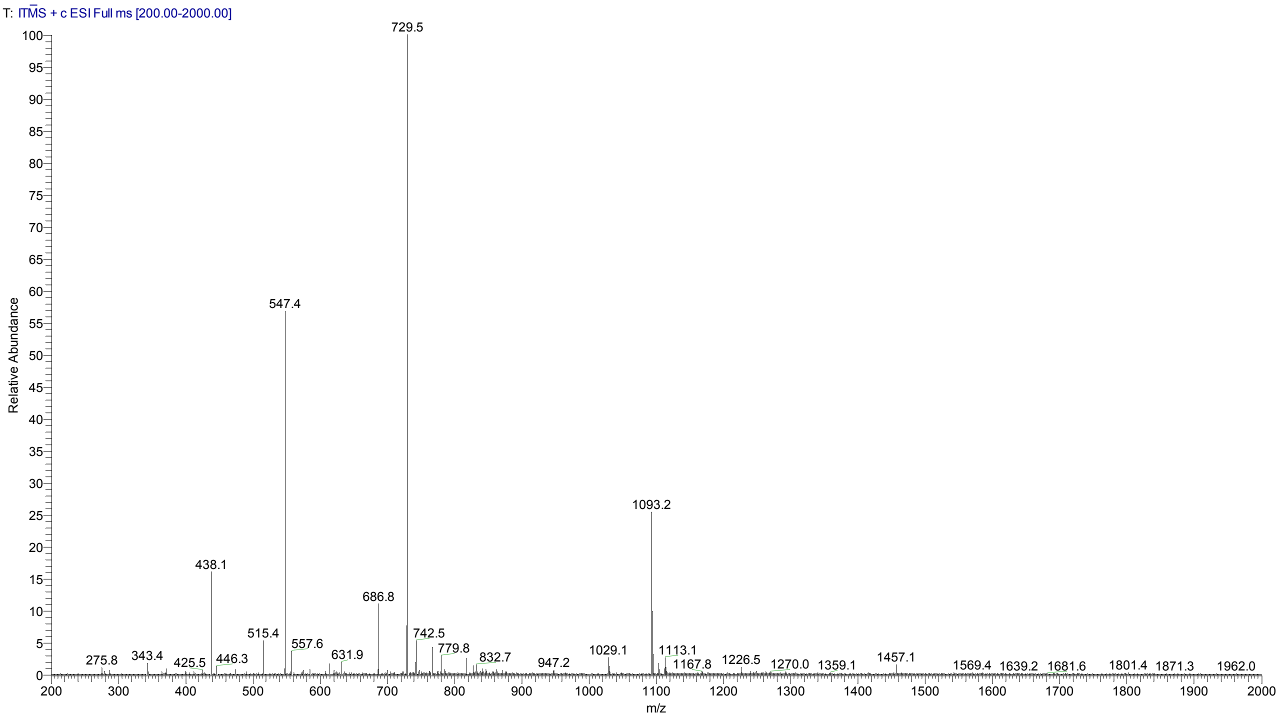

Figure S5. ESI Mass spectrum of Esc(1-21) (peptide **1**).

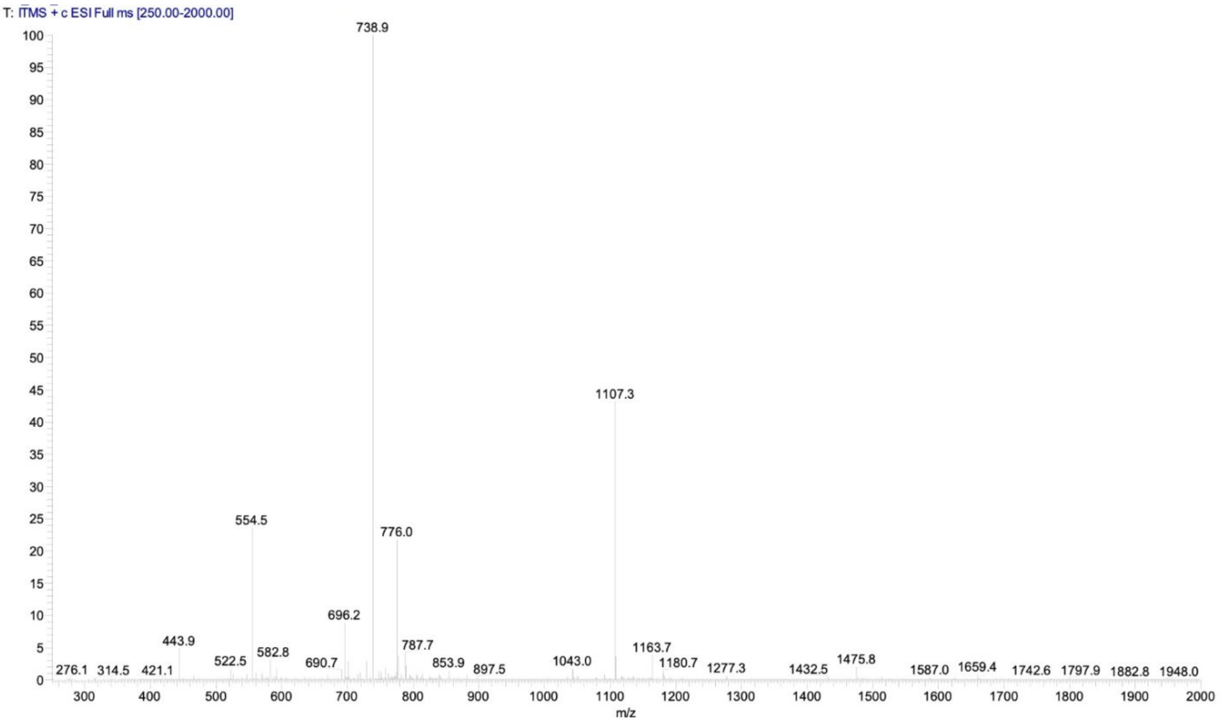

Figure S6. ESI Mass spectrum of [Aib^{8}]-Esc(1-21) (peptide **2**).

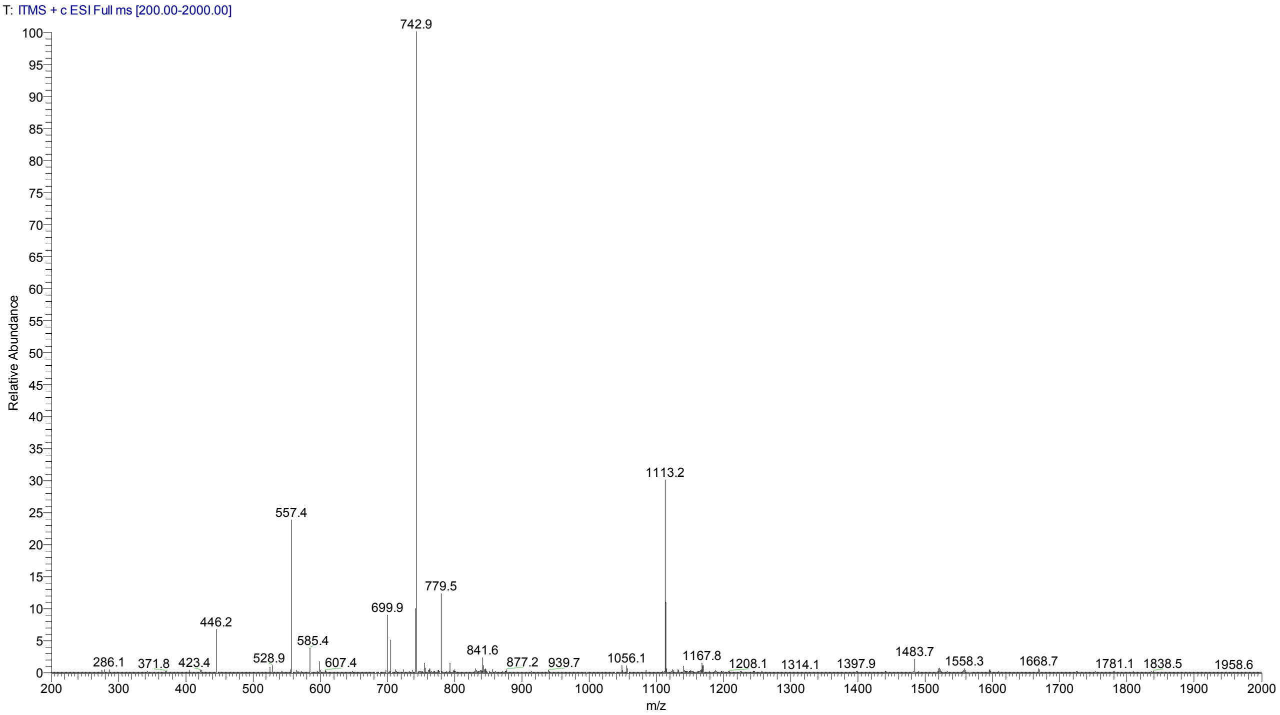

Figure S7. ESI Mass spectrum of [Pro^{8}]-Esc(1-21) (peptide **3**).

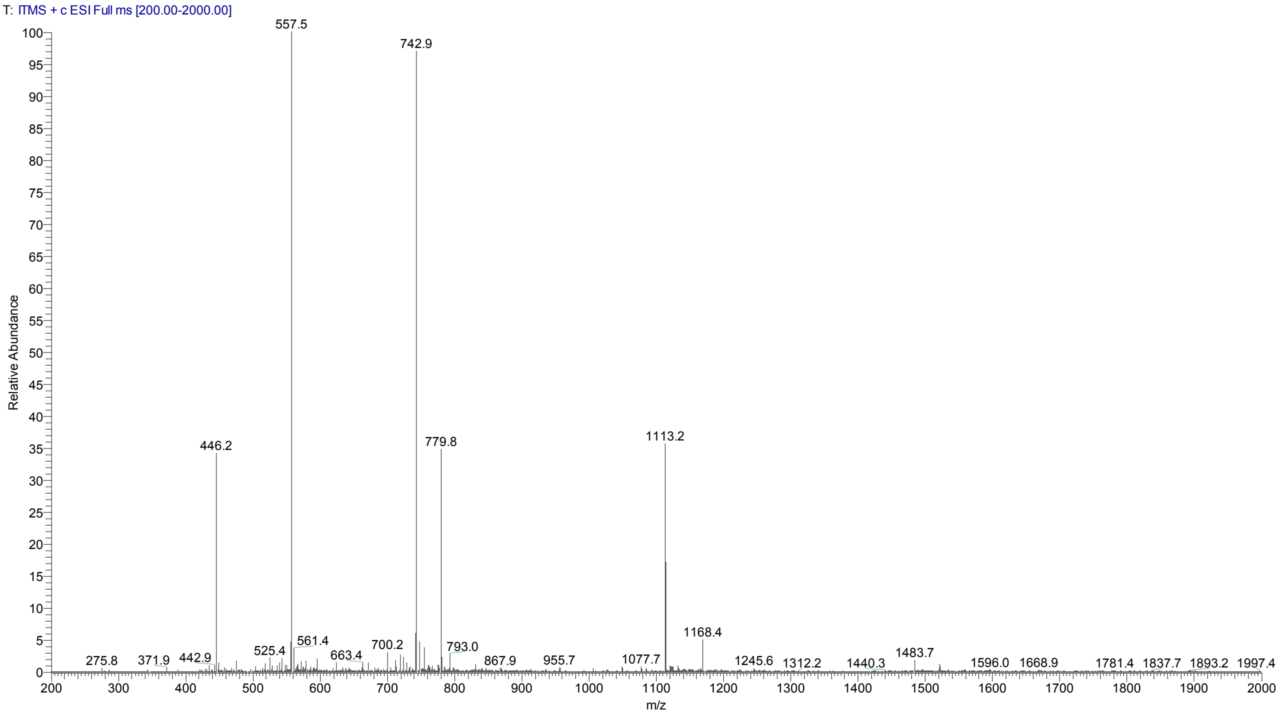

Figure S8. ESI Mass spectrum of [DPro^{8}]-Esc(1-21) (peptide **4**).

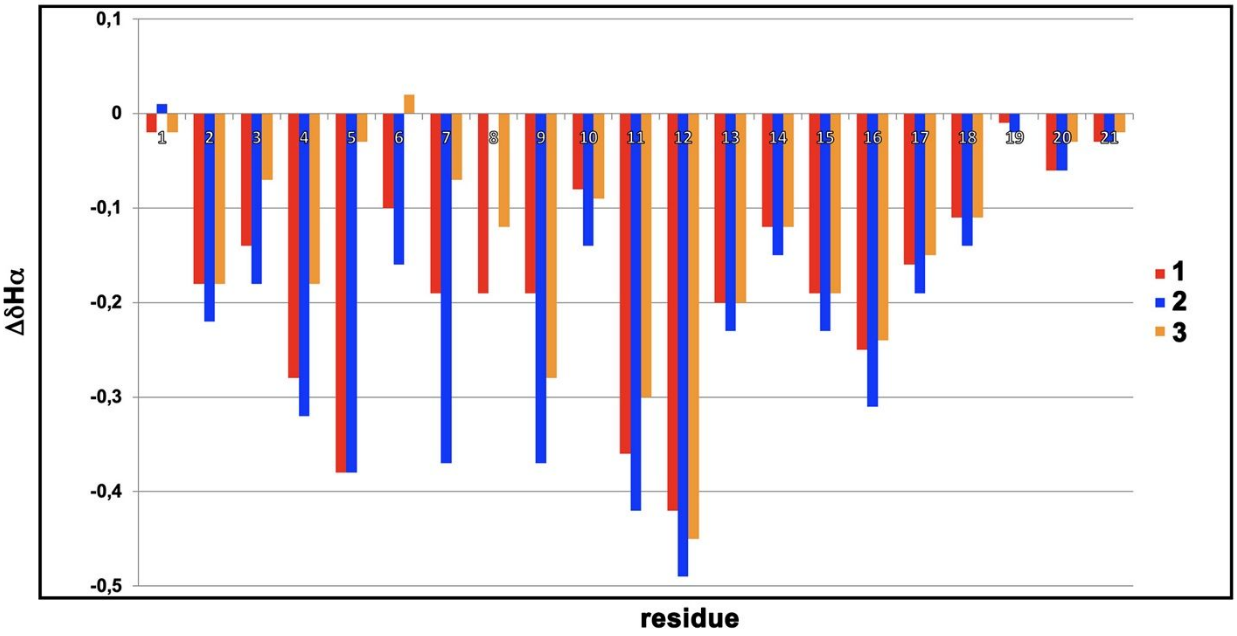

Figure S9. Plots of chemical shift deviations of Hα protons from random coil values for peptides **1**-**3** in bicelle solution. Random coil values were taken from ref: Andersen, N. H.; Liu, Z.; Prickett, K. S. Efforts toward deriving the CD spectrum of a 3(10) helix in aqueous medium. FEBS Lett. 1996, 399, 47-52.
